# Ethnicity and Child Health in Northern Tanzania: Maasai Pastoralists Are Disadvantaged Compared to Neighbouring Ethnic Groups

**DOI:** 10.1371/journal.pone.0110447

**Published:** 2014-10-29

**Authors:** David W. Lawson, Monique Borgerhoff Mulder, Margherita E. Ghiselli, Esther Ngadaya, Bernard Ngowi, Sayoki G. M. Mfinanga, Kari Hartwig, Susan James

**Affiliations:** 1 Department of Population Health, London School of Hygiene and Tropical Medicine, London, England, United Kingdom; 2 Department of Anthropology, University of California Davis, Davis, California, United States of America; 3 Savannas Forever Tanzania, Arusha, Tanzania; 4 University of Minnesota, Minneapolis, Minnesota, United States of America; 5 National Institute for Medical Research, Muhimbili Medical Research Centre, Dar es Salaam, Tanzania; 6 St. Catherine University, Minneapolis, Minnesota, United States of America; Indiana University, United States of America

## Abstract

The Maasai of northern Tanzania, a semi-nomadic ethnic group predominantly reliant on pastoralism, face a number of challenges anticipated to have negative impacts on child health, including marginalisation, vulnerabilities to drought, substandard service provision and on-going land grabbing conflicts. Yet, stemming from a lack of appropriate national survey data, no large-scale comparative study of Maasai child health has been conducted. Savannas Forever Tanzania surveyed the health of over 3500 children from 56 villages in northern Tanzania between 2009 and 2011. The major ethnic groups sampled were the Maasai, Sukuma, Rangi, and the Meru. Using multilevel regression we compare each ethnic group on the basis of (**i**) measurements of child health, including anthropometric indicators of nutritional status and self-reported incidence of disease; and (**ii**) important proximate determinants of child health, including food insecurity, diet, breastfeeding behaviour and vaccination coverage. We then (**iii**) contrast households among the Maasai by the extent to which subsistence is reliant on livestock herding. Measures of both child nutritional status and disease confirm that the Maasai are substantially disadvantaged compared to neighbouring ethnic groups, Meru are relatively advantaged, and Rangi and Sukuma intermediate in most comparisons. However, Maasai children were less likely to report malaria and worm infections. Food insecurity was high throughout the study site, but particularly severe for the Maasai, and reflected in lower dietary intake of carbohydrate-rich staple foods, and fruits and vegetables. Breastfeeding was extended in the Maasai, despite higher reported consumption of cow's milk, a potential weaning food. Vaccination coverage was lowest in Maasai and Sukuma. Maasai who rely primarily on livestock herding showed signs of further disadvantage compared to Maasai relying primarily on agriculture. We discuss the potential ecological, socioeconomic, demographic and cultural factors responsible for these differences and the implications for population health research and policy.

## Introduction

As we approach their 2015 target, there has been mounting criticism of the strong emphasis the Millennium Development Goals, and related development objectives, have placed on national averages, because such estimates dangerously obscure considerable and often rising inequality in health within developing countries [1]–[3]. Most scholarship focuses on socioeconomic dimensions of health (e.g. [4]), but, in some contexts, ethnic disparities can be of equal or greater importance [5], [1]. A particular concern is the status of minority populations, or of “indigenous” ethnic groups, which systematically fail to benefit from wider improvements in health experienced by the general population. This is especially true when such groups are geographically or linguistically remote, or when they rely on alternative modes of production to agriculture, which benefits selectively from national and international investments and technological innovation [6], [7], [8]. In this paper we investigate ethnic variation in child health in Northern Tanzania, focusing on the comparison of the Maasai to neighbouring ethnic groups, and furthermore between farming and pastoralist households among the Maasai.

Many researchers have emphasised the numerous difficulties faced by the Maasai, and by pastoralists more generally [9]–[13]. A number of Tanzanian non-governmental organisations (NGOs) also specifically address development issues in the Maasai, many of which are included in the Pastoralists and Indigenous NGOs Forum (www.pingosforum.or.tz), an advocacy coalition of over 50 NGOs. However, due to the lack of national data disaggregating health outcomes by ethnicity or livelihood, there has so far been no large-sample comparative assessment of the true extent to which the health of the Maasai can be considered disadvantaged relative to the wider population [12]. This lack of data is an important concern since such invisibility in aggregate datasets represents a crucial barrier to motivating both productive discourse and support for initiatives addressing the specific needs of disadvantaged and/or marginalised ethnic groups [7].

The Maasai people inhabit Tanzania and Kenya, and are often characterised as archetypal pastoralists i.e. subsistence is based primary or exclusively on livestock herding. However, specialised pastoralism, traditionally the core of Maasai cultural identity, declined throughout the twentieth century and today livelihoods are increasingly diversifying towards agro-pastoralism and off-farm activities [14], [15]. Owing to their proximity to the major East African game parks and their distinctive customs and dress, the Maasai have become perhaps the most globally recognizable ethnic group in sub-Saharan Africa [15]. Indeed they are commonly (mis)represented in advertisements and tourist commercials for both Kenya and Tanzania, and in recent years have become the focus of a burgeoning cultural tourism economy [16], [17].

There are a number of reasons to suspect that the fame of the Maasai is not matched with good fortune with respect to child health. First, the Maasai reside in a semi-arid ecology prone to erratic rainfall and periodic drought. The year we initiated data collection (2009) witnessed a particularly devastating drought leading to high levels of pasture depletion, which by some reports the Maasai describe as the worst drought in living memory [18]. Such vulnerability is likely to be reflected in high food insecurity and poor health outcomes. Second, Maasai communities tend to be relatively remote, and livestock herding requires most household units to be at least semi-nomadic. In contrast to other ethnic groups, they also have a relatively poor command of Swahili, the national language. These factors reduce opportunities for both health service provision and educational attainment [12]. Third, these disadvantages have been exacerbated by on-going land ownership conflicts, fuelled in large part by the expansion of parks and protected areas for the profitable ecotourism industry, causing the Maasai to be displaced from historical and often the most fertile rangelands [19], [20]. Such conflicts are anticipated to have both direct and indirect negative trickle-down consequences for child well being.

The question of ethnic variation in child health is also particularly interesting in Tanzania because, with an estimated 120 distinct ethnicities inhabiting its borders, it combines the highest level of ethnic diversity in Sub-Saharan Africa [21], with a tradition of downplaying ethnic differences in order to emphasize collective national identity. This stems from the socialist ideology that characterized the emergence of an independent Tanzania, and contrasts with Kenya, where ethnicity is highly politicized [22], [23]. While playing a relatively modest role in national politics, ethnic identity (locally referred to as “tribe” or “kabila” in Kiswahili) is widely recognised and remains closely associated with region of residence, and linguistic and cultural distinctions, see Spear and Waller [15] for a specific discussion of the construction and expression of Maasai ethnic identity. Despite this diversity, ethnicity data are not routinely collected in national surveys. Five Tanzanian Demographic and Health Surveys (DHS), the only source of nationally representative data on child health, have so far been conducted (1991/2, 1996, 1999, 2004/5, 2010). To our knowledge, only the 1991/2 and 1996 DHS provide ethnicity data and such information has never been used to consider ethnic variation in child health.

We are aware of only a handful of previous studies that explicitly address ethnic variation in child health in Tanzania [24]–[27]. Hadley [26], for example, in a sample of several hundred children living in the Rukwa valley, reports that Sukuma children had significantly lower mortality and superior nutritional status compared to the Pimbwe. Unable to account for these differences in terms of (measured) variation in wealth or seasonal food insecurity, Hadley speculates that different infant feeding practices accounted for observed differences, see also [28], [29]. In a large sample, Kruger et al. [27] reported that Datoga pastoralists had significantly lower child immunisation coverage compared to other ethnicities resident in the Mbulu area of northern Tanzania, a pattern they attribute to the relatively remote Datoga being both less accessible to and less trusting of health services. Numerous small-scale studies have also measured child survival and health within specific ethnic groups (e.g. Datoga: [30], [31]; Pwimbwe and Sukuma: [26], [32]; Maasai: [33]; Hadza: [34]). However, comparing estimates across studies is problematic due to differences in sampling methodology, timing, statistical methodology, and health measures considered.

In this study we conduct a systematic analysis of ethnic variation in child health using data collected by Savannas Forever Tanzania (SFTZ), an NGO specialising in the evaluation of rural development projects. SFTZ surveyed 56 villages spanning seven administrative regions in northern Tanzania between 2009 and 2011 as part of the Whole Village Project (WVP). Substantial sampling in the Arusha region and surrounding area lead to the inclusion of large number of Maasai households, making the collected data a uniquely valuable resource for the study of the Maasai relative to neighbouring ethnic groups. For comparison, in the 1996 Tanzanian DHS only 2% (n = 195/8120) of sampled women identified as Maasai [35]: p77–78, compared to 21% (n = 735/3584) of SFTZ sampled households. The main other ethnic groups sampled by SFTZ were the Sukuma, Rangi and Meru. All of these ethnicities are Bantu ethno-linguistic groups and, in contrast to the predominantly pastoralist and Nilotic-speaking Maasai, their livelihoods are typically based on agro-pastoralism or small-scale agriculture. The Sukuma are the largest ethnic group in Tanzania by a clear margin, estimated to represent 16% of the national population, while no other ethnicity comprises more than 5% [35]: p77–78.

Following the literature described above, we hypothesize that child health will be poorest in Maasai communities relative to neighbouring ethnic groups, and particularly for those Maasai who rely primarily on livestock herding. We contextualize our analyses by presenting descriptive data on household socioeconomic, demographic and cultural characteristics by ethnicity, but do not attempt to isolate the role of these more distal factors via multivariate analysis. Instead our focus here is on establishing a systematic and detailed characterisation of the nature of ethnic disparities in health across a large range of measures. Such information is valuable to the design and implementation of health policies and initiatives that typically focus on specific health risks and proximate determinants (e.g. nutrition, breastfeeding, vaccination), but rarely consider ethnic variation. Furthermore, since a number of development organisations in Tanzania focus specifically on Maasai communities or on pastoralists more generally, our data provide a much-needed clarification of the extent to which such groups can be meaningfully understood to be disadvantaged with regard to child health.

In the analyses that follow, we compare each ethnic group on the basis of (**i**) measures of child health, including anthropometric indicators for nutritional status, subjective health ratings and the self-reported incidence of specific illnesses. We also compare these groups on (**ii**) several important proximate determinants of child health, including food insecurity, diet, breastfeeding and vaccination coverage. We then (**iii**) further contrast households among the Maasai by the extent to which they rely on livestock herding as their primary livelihood. For the purposes of the present study, we do not conceptualize ethnicity as a causal determinant of child health, nor do we attempt to analytically isolate ethnic differences that are independent from socioeconomic or demographic variation. Our objective is simply to quantify disparities in child health on the basis of self-reported ethnicity (see below); disparities that may ultimately originate from a complex suite of interrelated determinants. We do however include a supplementary analysis of the contribution of a limited number of ecological factors (village rainfall, distance to district capital and presence/absence of health clinics/dispensaries) to village-level differences in child anthropometric indicators. The implications of our results for both policy makers and population health scientists are discussed.

## Materials and Methods

### The Whole Village Project

**Figure 1:**
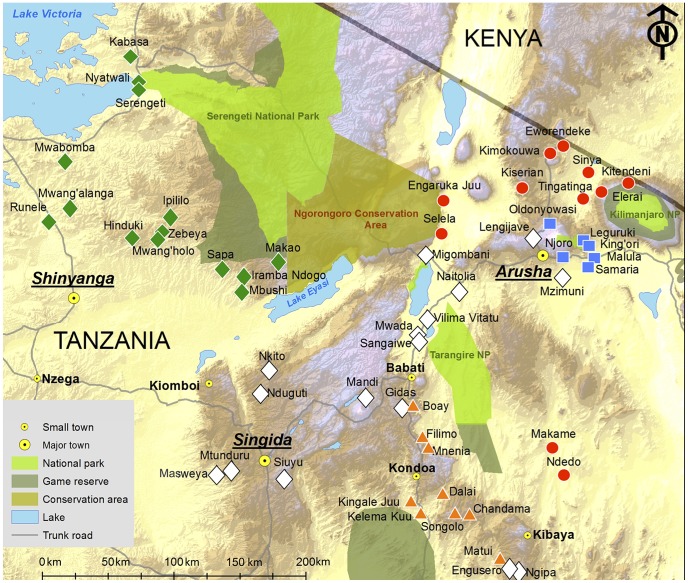
of the 56 Study Villages Included in the Whole Village Project. Ethnicity is coded as the most common ethnicity in each village (**File S1** for details). Red circle  =  Maasai village; orange triangle  =  Rangi village; green diamond  =  Sukuma village; blue square  =  Meru village; white diamond  =  Other ethnicity village. Figure adapted from Aichele et al. [76] and initially published under an open-access license (CC-BY).

All data come from the WVP, a project run by SFTZ and the University of Minnesota, and in collaboration with the National Institute for Medical Research (NIMR), Muhimbili Medical Research Centre in Tanzania. The purpose of the WVP is to provide baseline data for evaluating rural development projects in the region, as well as a general source of information on Tanzanian villages. Overall, 56 villages were sampled, between mid 2009 and mid 2011, across the northern and central regions of Arusha (19 villages), Manyara (11 villages), Dodoma (7 villages), Singida (5 villages), Shinyanga (8 villages), Mwanza (3 villages), and Mara (3 villages). The sampling of villages was based in part on the priorities of development agency partners and the permission of government leaders, although effort was made to randomize village sampling where possible and to ensure a wide geographic spread. SFTZ and WVP have no agenda with regard to Maasai settlement or pastoralist rights, and village sampling was not influenced by the ethnicity or livelihood of residents. **Figure1** shows the location of each village in relation to major settlements, main roads, national parks and game reserves.

The WVP collected quantitative and qualitative data at the level of village, household and child. This study primarily utilises quantitative data from the household and child level. Within each village between 60–75 households were randomly selected for participation from a list provided by village administrators, leading to a total of 3584 surveyed households. Household heads responded to questionnaire modules surveying their demographic, cultural and socioeconomic characteristics. Within each household, a child health module was administered collecting anthropometric and questionnaire data for all eligible children under the age of five years. More information on the WVP, including descriptive statistics on village characteristics by district, is available on the SFTZ website (sftz.org) or by request to the first author. All data utilized in this study are available from SFTZ to interested researchers upon request, and following approval from the SFTZ board of directors (see sftz.org).

### Ethics Statement

Informed oral consent was obtained from participants and all individual data were anonymized before analysis. Consent was oral rather than written because this format is most appropriate in rural Tanzanian communities with limited literacy skills, and where many individuals harbour mistrust of written communication. Participant consent was recorded in separate documentation. The WVP, including consent procedures, received ethical approval from the University of Minnesota (Institutional Review Board code number: 0905S65241) and from the National Health Research Ethics Review Committee, at NIMR.

### Ethnicity and Livelihoods

The study area is ethnically diverse, but four main ethnicities, the Maasai, Sukuma, Rangi and Meru, make up 60% of the households sampled and 65% of the households contributing child health data. Maasai (21% of households) were primarily sampled in the Arusha and Manyara regions. Sukuma (20% of households) were primarily sampled in the Mwanza and Shinyanga regions. Rangi (12% of households) were primarily sampled in the Dodoma region. Meru (8% of households) were primarily sampled in the Arusha region. Ethnicity was self-reported by the household head as a freeform response to the question “What is your tribe?” (“kabila” in Kiswahili). Most villages tended to be largely ethnically homogenous i.e. most households sampled stated the same tribal identity, particularly for villages in which the Maasai or Sukuma were most common (typically 85%+) and to a lesser extent for Meru and Rangi villages. However, many Maasai (12%) and Sukuma households (10%) were located in villages where they were not the majority ethnic group. Supporting information in **File S1** provides further details of the sampling by ethnicity, village, district and region. **File S2** and **File S3** provide supporting information on household and village-level data.

For the purpose of this study, livelihood categorization was based on self-reported primary livelihood strategy rather than a quantified behavioural measure. Around two-thirds of Maasai (68%) stated livestock herding as their “primary occupation”, while a quarter stated farming, the most common occupation for all other ethnicities. A small percentage of households stated their occupation as business, most commonly in the Meru (11%). We make the assumption that stated occupation serves as an effective proxy for primary mode of subsistence. We note however that many households identifying their primary livelihood as “farming” also kept livestock (i.e. agro-pastoralists), and that with recent trends in pastoralist livelihood diversification, some Maasai may still identify themselves as livestock herders despite considerable reliance on farming or wage-labour. Indeed, around half of Maasai households who identified their primary occupation as livestock herding reported cultivating at least some land.

### Child Health Data

Out of 3584 sampled households, 2268 (63%) contributed data on child health for children under five years of age, and just under half of those households provided data on more than one child (2 children: 35%; 3+ children: 10%), leading to a total of 3586 surveyed children. The mean age of sampling was 28.9 months, with roughly even sampling across the age range of zero to 60 months (**SI4**). An ANOVA showed a significant difference in mean ages of sampled children by ethnicity (F (4,3581)  = 3.09, p<0.05). The largest difference was very small in magnitude with Sukuma children sampled at a mean age of 27.8 months compared to the Rangi at 31.3 months. There was no statistically significant difference in the sampling of boys vs. girls (51% female) across ethnicities (chi-squared (4,3586)  = 0.50, p>0.05).

Child weight was measured to the nearest 100 g using a Salter-type spring hanging scale for infants, and electronic scales for children able to stand. Child height was measured to the nearest millimetre using a measuring board for young children, and using a stadiometer for children of two years or older. All measurements were made once and immediately entered into a database. Children were measured by different field staff depending on the village sampled, but training of enumerators by UNICEF staff and oversight of anthropometric sessions by NIMR personnel ensured high levels of inter-rater reliability prior to data collection.

Three anthropometric indicators were derived using World Health Organisation (WHO) age and sex-specific growth standards [36] Height-for-age Z-scores (HAZ) serves as an indicator of long-term effects of malnutrition. A child with a HAZ of <−2 standard deviations from the WHO reference is considered “stunted” i.e. chronically malnourished, which reflects failure to receive adequate nutrition over a long period of time and is influenced by recurrent and chronic illness. Weight-for-height Z-scores (WHZ) measure body mass in relation to body height/length and describes current nutritional status. A child with a WHZ <−2 standard deviations is considered acutely malnourished (i.e. “wasted”), which represents the failure to achieve adequate nutrition in the period immediately preceding measurement and may result from inadequate food intake or illness. Weight-for-age Z-scores (WAZ) can be considered as a composite index, taking into account both chronic and acute malnutrition. Children with WAZ <−2 standard deviations are classified as “underweight”. Following WHO guidelines (**File S4**), extreme values, potentially resulting from measurement error, were removed leading to 3411, 3426 and 3507 valid HAZ, WHZ and WAZ scores respectively.

In all villages, carers were asked to categorize the health of each child as either “good” or “frequently sick”, and whether or not the child had *ever* suffered from the following illnesses/symptoms: fever, diarrhoea, pneumonia, cough/flu, malaria or worms. In 37/56 villages an additional question asked whether each illness/symptom had been experienced *in the last 3 months*. Recognizing the potential for recall bias when reporting childhood symptoms over a long period, particularly in older children, priority is given to the latter short-term version of this question in the present analysis. Locally recognisable terms for each illness/symptom were used following consultation with medical practitioners and NIMR staff.

Household and child-level surveys carried out in all villages also asked a number of questions relating to important proximate determinants of child health. First, the Household Food Insecurity Access Scale (HFIAS), was used to assess the extent to which households experienced problems assessing food during the last 30 days [37]. The HFIAS has previously been validated as a useful survey tool in rural Tanzania [38] and is described in more detail in the supplementary material (**File S2**). Second, food consumption was also measured by a diet survey recording the foods eaten by each child in the previous day divided into nine food group categories for analysis. The list of food items (see **File S4**) was constructed on the basis of a review of previous studies and prior field experience by the authors, and was piloted successfully as an inclusive dietary measure. Surveys did not record the amount of each food eaten, only whether or not some of each food was consumed. Third, enumerators recorded whether or not the carer stated the child was currently breastfeeding and/or eating solids, and whether the child had consumed colostrum in early infancy. Finally, survey respondents were asked whether or not the child had received recommended vaccinations (BCG (tuberculosis), Polio, DPT and Measles) and vitamin A supplementation. **File S4** provides further information on child-level data.

### Data Analysis

To address ethnic variation in child health we use multilevel linear and logistic regression models for continuous and dichotomous dependent variables respectively, accounting for the clustering of child-level data (level 1, n = 3568) within villages (level 2, n = 56) using random intercepts. Comparisons on the HFIAS are made at the household (n = 2268) rather than child-level, with multilevel logistic regression predicting whether or not a household is categorized as “severely food insecure”. Multilevel modelling is the appropriate statistical technique because failure to adjust estimates for non-independent sampling inflates the risk of Type 1 error. Furthermore, multilevel analysis quantifies unexplained variance at each hierarchical level, thus enabling us to assess the extent to which ethnicity accounts for variance in child health within and between villages. The average village provides data on 64 children from 41 households. In our child health analyses we do not include an intermediate hierarchical level for household because the mean number of children per household was only 1.58 and Clarke [39] has demonstrated that when clusters are unbalanced and sparsely populated (i.e. ≤2 cases per level) both fixed and random effects may be overestimated. All models were fit using maximum likelihood estimation in Stata version 13 using the “xtmixed” and “xtmelogit” commands [40]

For each outcome, we first contrast the four main ethnic groups, and then contrast households *among the Maasai* that self report as either farmers or livestock herders. All analyses adjust for effects of child age, child age-squared and child sex. We also adjust all analyses for the timing of village surveys in order to take into account whether or not the village was sampled in the hunger season (see **File S2**). Note that livelihood comparisons are derived from separate models with cases restricted to Maasai farming and pastoralist households only (715 Maasai children, from 21 villages). Sample size was also reduced for models estimating the estimation of child symptoms over the past 3 months. These comparisons are based on 2496 children from 37 villages when contrasting all ethnicities, and on 282 children from 8 villages when contrasting farming vs. pastoralist households among the Maasai.

## Results

### Household and village characteristics by ethnic group

**Table 1** summarises household and village level characteristics by ethnicity, and by livelihood among the Maasai. Dramatic socioeconomic differences between ethnic groups are apparent. For example, 68% of Maasai male household heads had no formal education whatsoever; while among the Meru 84% of male household heads had completed at least primary education. A wealth index, based on non-livestock asset ownership (**File S2**) indicates the Maasai had on average the lowest material wealth, while the Meru are the wealthiest. With regard to household structure, Sukuma households were the largest, and contained the most young children, while Meru were the smallest and contained the least young children. Maasai households contained the highest proportion of household heads in a polygynous marriage (40%), and the highest proportion of female-headed households (39%). Polygyny was also relatively common in the Sukuma and Rangi, but almost entirely absent among the Meru. Christianity (Catholic or Protestant) was the stated religion of two-thirds of Maasai, and around one half of Sukuma, who otherwise stated they practiced “traditional” religions. Almost all Rangi were Muslim, and almost all Meru were Protestant. Village-level data (see also **File S3**) confirm that Maasai pastoralists were resident in the driest villages overall (625 mm per year), while Meru households lived in the wettest villages (982 mm per year). Meru households were less remote compared to all other ethnic groups, and most often resided in villages with a health dispensary or clinic, compared to all other ethnic groups. Two-thirds of Maasai farmer households lived in a village with a health clinic or dispensary compared to just over half of Maasai pastoralist households.

**Table 1:** Descriptive Statistics for Households Contributing Child Health Data by Ethnicity.

		MAASAI						
			*Primary occupation*					
		All	*Livestock*	*Farming*	SUKUMA	RANGI	MERU	OTHER
*No. of villages where ethnic group is the majority* (n = 56)		11	-	-	14	9	6	16
*No. of households contributing child health data* (n = 2268)		542	366	124	517	240	165	804
*No. of children sampled* (n = 3586)		788	523	192	1025	354	213	1206
**Household-Level**								
Primary occupation of household head	Livestock Herding (%)	68	100	-	<1	<1	3	5
	Farming (%)	23	-	100	92	95	73	82
	Business (%)	3	-	-	4	3	11	7
	Other (%)	6	-	-	4	2	13	6
Education level (male household heads only)	None (%)	68	70	61	27	19	4	21
	<Standard 7 (%)	6	5	8	18	18	11	14
	Standard 7+ (%)	26.4	25	31	55	64	84	65
Mean wealth index		1.8 *[1.5]* a	1.6 *[1.4]*	2.2 *[1.5]*	3.0 *[1.5]*	3.3 *[1.6]*	5.6 *[2.5]*	3.4 *[2.1]*
Mean household size		5.8 *[2.3]*	5.7 *[4.2]*	6.3 *[2.4]*	8.4 *[3.9]*	6.2 *[2.1]*	5.6 *[1.7]*	6.3 *[2.3]*
Mean no. of children per household		1.5 *[0.8]*	1.5 *[0.8]*	1.5 *[0.8]*	2.0 *[1.1]*	1.4 *[0.7]*	1.2 *[0.5]*	1.5 *[0.7]*
Household type	Polygynously Married Household Head (%)	40	44	34	26	16	2	16
	Female-Headed Household (%)	39	42	31	18	16	10	16
Religion	Protestant (%)	48	44	52	24	<1	98	44
	Catholic (%)	21	23	21	22	3	1	22
	Muslim (%)	<1	<1	<1	3	97	<1	22
	Traditional/Other (%)	30	32	27	52	<1	<1	12
**Village-Level**								
Mean Village Annual Rainfall (mm)		653 *[132]*	625 [*64*]	727 *[213]*	841 [*68*]	688 [*39*]	982 *[121]*	779 *[187]*
Mean Village Distance from district capital (km)		32.1 *[18.9]*	30.7 *[18.1]*	36.1 *[21.2]*	34.3 *[17.7]*	33.2 *[13.8]*	21.3 *[17.5]*	33.0 *[17.0]*
Village has own health dispensary/clinic? (% yes)		54	53	66	56	52	70	59

a Numbers in square brackets are standard deviations.

### Are there ethnic differences in child health?

**Tables 2** **–** **6** present unadjusted descriptive statistics for each dependent variable by ethnicity, and by livelihood among the Maasai, along with fixed effects coefficients from corresponding multivariate multilevel regression models, which are adjusted for village-level clustering (i.e. using random intercepts), along with fixed effects of child age, child age squared, child sex and season of data collection. **File S5** reports the fixed effects for all covariates in each model, along with random effects estimating the unexplained variance within and between villages before and after models have been adjusted for ethnicity.

**Table 2:** Multilevel Linear Regressions Predicting Child Anthropometric Status.

		Height for Age Z Score (n = 3411)			Weight for Height Z Score (n = 3426)			Weight for Age Z Score (n = 3507)		
		Mean (SD)	% stunted	Adjusted B coefficient (95% CI)	Mean (SD)	% wasted	Adjusted B coefficient (95% CI)	Mean SD	% underweight	Adjusted B coefficient (95% CI)
Ethnicity	Maasai	−2.13 (1.78)	57	0.00	−0.20 (1.60)	10	0.00	−1.38 (1.48)	33	0.00
	Sukuma	−1.34 (1.41)	32	**0.59** *** *(0.36–0.82)*	0.40 (1.20)	2	**0.44** *** *(0.26–0.63)*	−0.47 (1.15)	7	**0.72** *** *(0.55–0.89)*
	Rangi	−1.83 (1.34)	44	0.22 *(*−*0.05–0.49)*	−0.07 (1.12)	3	0.04 *(*−*0.18–0.27)*	−1.06 (1.07)	17	**0.27** * *(0.06–0.47)*
	Meru	−0.93 (1.37)	21	**0.92** *** *(0.60–1.25)*	0.38 (1.36)	3	**0.43** ** *(0.16–0.70)*	−0.26 (1.17)	5	**0.87** *** *(0.62–1.12)*
	Other	−1.69 (1.56)	43	**0.32** ** *(0.13–0.51)*	0.18 (1.26)	3	**0.26** ** *(0.10–0.41)*	−0.80 (1.20)	13	**0.46** *** *(0.31–0.60)*
Livelihood a (*Maasai only*)	Livestock	−2.21 (1.75)	59	0.00	−0.25 (1.62)	12	0.00	−1.44 (1.46)	35	0.00
	Farmer	−1.84 (1.84)	51	**0.39** * *(0.06–0.71)*	−0.02 (1.63)	6	0.20 *(*−*0.09–0.48)*	−1.12 (1.55)	27	**0.39** ** *(0.13–0.65)*

Adjusted B coefficients are adjusted for child age, child sex, hunger season and a random intercept for village.

*p<0.05,

**p<0.01,

***p<0.001,

a Livelihood parameters are derived from separate models including only Maasai households.

**Table 3:** Multilevel Logistic Regressions Predicting Subjective Health and Self-Reported Incidence of Specific Illnesses/Symptoms.

		Subjective Health (n = 3585)		“*Has the Child Had X in the Past Three Months?*” *(n = 2496)*											
		“Good” vs. “Frequently Sick”		Fever		Diarrhoea		Pneumonia		Cough/Flu		Malaria		Worms	
		% sick	Adjusted Odds Ratio (95% CI)	% yes	Adjusted Odds Ratio (95% CI)	% yes	Adjusted Odds Ratio (95% CI)	% yes	Adjusted Odds Ratio (95% CI)	% yes	Adjusted Odds Ratio (95% CI)	% yes	Adjusted Odds Ratio (95% CI)	% yes	Adjusted Odds Ratio (95% CI)
Ethnicity	Maasai	22	1.00	55	1.00	30	1.00	12	1.00	53	1.00	18	1.00	5	1.00
	Sukuma	13	**0.58** ** *(0.40–0.84)*	58	1.12 *(0.82–1.52)*	32	1.00 *(0.67–1.50)*	4	0.48 *(0.18–1.28)*	58	1.32 *(0.94–1.87)*	24	1.26 *(0.79–2.03)*	6	1.37 *(0.62–3.01)*
	Rangi	19	0.90 *(0.58–1.38)*	46	0.70 *(0.48–1.03)*	27	0.93 *(0.56–1.52)*	3	**0.31** * *(0.10–0.96)*	63	1.40 *(0.92–2.11)*	33	**1.77** * *(1.03–3.07)*	6	1.05 *(0.41–2.71)*
	Meru	6	**0.24** *** *(0.12–0.48)*	37	**0.48** ** *(0.30–0.77)*	10	**0.28** ** *(0.13–0.60)*	3	0.37 *(0.10–1.39)*	56	1.17 *(0.71–1.93)*	22	1.06 *(0.54–2.05)*	17	**3.27** * *(1.24–8.6)*
	Other	17	0.78 *(0.57–1.06)*	46	**0.70** * *(0.51–0.94)*	25	0.76 *(0.51–1.14)*	4	**0.43+** *(0.18–1.02)*	54	1.05 *(0.75–1.45)*	27	1.43 *(0.91–2.27)*	7	1.24 *(0.57–2.69)*
Livelihood a (*Maasai only*)	Livestock	23	1.00	52	1.00	33	1.00	12	1.00	52	1.00	16	1.00	2	1.00
	Farmer	18	0.71 *(0.44–1.15)*	63	**1.74** * *(1.04–2.91)*	24	0.63 *(0.36–1.12)*	11	1.00 *(0.45–2.23)*	59	1.20 *(1.45–3.21)*	20	1.51 *(0.78–2.89)*	9	**4.91** * *(1.41–17.04)*

Adjusted odds ratios are adjusted for child age, child sex, hunger season and a random intercept for village.**+** p<0.1,

* p<0.05,

**p<0.01,

***p<0.001,

a Livelihood parameters are derived from separate models including only Maasai households.

**Table 4:** Multilevel Logistic Regression Predicting Household Food Insecurity (n = 2208).

		% Food Secure	% Mildly Food Insecure	% Moderately Food Insecure	% Severely Food Insecure	Adjusted Odds Ratio Predicting Severe Food Insecurity (95% CI)
Ethnicity	Maasai	4.7	2.1	15	78	1.00
	Sukuma	8	4.9	55	32	**0.18** *** *(0.12–0.28)*
	Rangi	6	6	41	47	**0.32** *** *(0.20–0.51)*
	Meru	12	12	53	24	**0.13** *** *(0.07–0.24)*
	Other	11	7	46	36	**0.20** *** *(0.14–0.29)*
Livelihood a (*Maasai only*)	Livestock	3.9	1.7	14	81	1.00
	Farmer	5	4.0	19	72	0.78 *(0.41–1.49)*


Adjusted odds ratios are adjusted for hunger season and a random intercept for village. See File SI2 for details on the Household Food Insecurity Access Scale.

***p<0.001,

a Livelihood parameters are derived from separate models including only Maasai households.

**Table 5:** Multilevel Logistic Regressions Predicting Breastfeeding Behaviour.

		Baby Had Colostrum		Currently Breastfeeding		Currently Eating Solids	
		(n = 3530)		(n = 3195)		(n = 3586)	
		% yes	Adjusted Odds Ratio	% yes	Adjusted Odds Ratio	% yes	Adjusted Odds Ratio
			(95% CI)		(95% CI)		(95% CI)
Ethnicity	Maasai	98	1.00	44	1.00	85	1.00
	Sukuma	91	**0.22** *** *(0.10–0.48)*	30	**0.15** *** *(0.09–0.25)*	90	1.76 *(0.81–3.85)*
	Rangi	96	0.73 *(0.27–1.99)*	29	**0.27** *** *(0.14–0.51)*	95	2.07 *(0.78–5.47)*
	Meru	99	2.53 *(0.49–13.11)*	31	**0.41** * *(0.20–0.84)*	84	0.77 *(0.26–2.25)*
	Other	97	0.58 *(0.28–1.22)*	32	**0.27** *** *(0.18–0.41)*	88	1.45 *(0.78–2.69)*
Livelihood a	Livestock	99	1.00	45	1.00	84	1.00
(*Maasai only*)	Farmer	97	0.50 *(0.14–1.70)*	37	0.77 *(0.46–1.30)*	85	1.43 *(0.70–2.92)*

Adjusted odds ratios are adjusted for child age, child sex, hunger season and a random intercept for village.

*p<0.05,

**p<0.01,

***p<0.001,

a Livelihood parameters are derived from separate models including only Maasai households.

**Table 6:** Multilevel Logistic Regressions Predicting Vaccination Coverage and Vitamin A Supplementation.

		BCG Vaccination (n = 3586)		Polio Vaccination (n = 3586)		DPT Vaccination (n = 3586)		Measles Vaccination (n = 3586)		Vitamin A Supplementation (n = 3586)	
		%	Adjusted Odds	%	Adjusted Odds Ratio	%	Adjusted Odds Ratio	%	Adjusted	%	Adjusted Odds Ratio
		yes	Ratio	yes		yes		yes	Odds Ratio	yes	
Ethnicity	Maasai	95	1.00	95	1.00	93	1.00	76	1.00	77	1.00
	Sukuma	94	1.38 *(0.64–2.99)*	94	1.49 *(0.73–3.03)*	92	1.34 *(0.70–2.62)*	67	0.84 *(0.50–1.40)*	60	0.75 *(0.44–1.25)*
	Rangi	97	1.94 *(0.68–5.54)*	97	2.54 *(0.94–6.89)*	96	**2.56** * *(1.03–6.35)*	82	**2.02** * *(1.08–3.80)*	76	1.03 *(0.58–1.83)*
	Meru	98	2.01 *(0.49–8.27)*	99	**4.50** * *(1.07–18.81)*	96	1.93 *(0.69–5.32)*	86	**2.66** * *(1.22–5.83)*	89	**2.43** * *(1.15–5.13)*
	Other	95	1.25 *(0.66–2.37)*	96	1.43 *(0.79–2.59)*	95	1.49 *(0.87–2.56)*	78	**1.58** * *(1.03–2.41)*	76	1.26 *(0.84–1.90)*
Livelihood a (*Maasai only*)	Livestock	96	1.00	95	1.00	94	1.00	77	1.00	78	1.00
	Farmer	92	*0.50 (0.22–1.15)*	94	0.85 *(0.39–1.86)*	92	0.76 *(0.36–1.62)*	76	1.17 *(0.66–2.07)*	76	1.24 *(0.68–2.26)*

Adjusted odds ratios are adjusted for child age, child sex, hunger season and a random intercept for village.

*p<0.05,

a Livelihood parameters are derived from separate models including only Maasai households.

**Figure 2:**
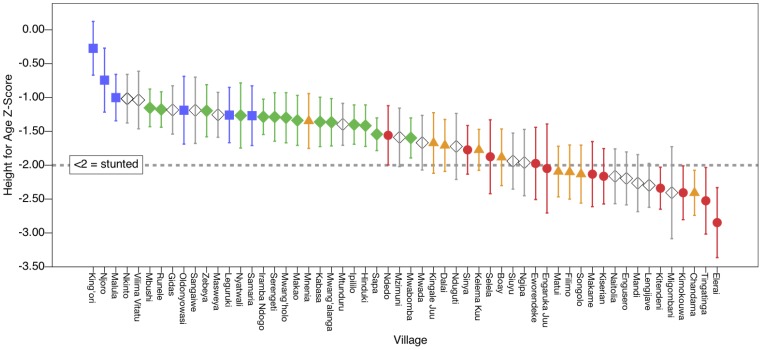
Height for Age Z-Score (HAZ) by Village and Ethnicity. Village means arranged in descending order, with 95% confidence intervals. Ethnicity is coded as the most common ethnicity in each village (see **File S1** for details). Children in Maasai majority villages tend to have relatively low HAZ scores and children in Meru majority villages tend to have relatively high HAZ scores, with Sukuma and Rangi majority villages intermediate between these extremes. Red circle  =  Maasai village; orange triangle  =  Rangi village; green diamond  =  Sukuma village; blue square  =  Meru village; white diamond  =  Other ethnicity village. HAZ scores falling below <2 are categorized as “stunted” by the World Health Organization.

There are striking ethnic differences in child nutritional status as indicated by anthropometric measurements (**Table 2**). Maasai children are at a considerable disadvantage, while Meru children are at a relative advantage. Nearly three times as many Maasai children are stunted (57%) compared to Meru children (21%), and almost double as many when compared to Sukuma children (32%). Rangi children had a level of stunting intermediate between the Sukuma and Maasai (44%). In terms of adjusted HAZ values, the contrast between Maasai and Meru children corresponds to almost a full standard deviation (B = 0.92, 95% Confidence Interval (CI)  = 0.60–1.25, p<0.001). Wasting showed a similar pattern, with more than three times as many Maasai children recorded as wasted (10%) compared to any other ethnic group (2–3%). Adjusted WHZ scores show these differences correspond to around half a standard deviation when contrasting the Maasai to the Sukuma or Meru (B = 0.44, 95%CI = 0.26–0.63, p<0.001; and B = 0.43, 95%CI = 0.16–0.70, p<0.001 respectively), but the comparison did not reach statistical significance when contrasting the Maasai to the Rangi. WAZ similarly places Maasai children as having the worst, and Meru the best, nutritional status compared to other ethnic groups. Random effects indicate that 5–10% of variance in child anthropometric status is between rather than within villages, and that adding ethnicity into each model greatly reduces the amount of unexplained between-village variance (**File S5**). **Figure2** illustrates the substantial village and ethnic variation in anthropometric status, displaying the means of HAZ for each village coded by the most common resident ethnicity.

There are also ethnic differences in subjective health and self-reported incidence of symptoms/illnesses in the three months prior to the survey (**Table 3**). Overall 17% of children were described as frequently sick by their carers. Compared to the Maasai, Rangi children were not significantly different on this measure. However the odds of being described as frequently ill was 42% lower for the Sukuma (Odds Ratio (OR)  = 0.58, 95%CI = 0.40–0.84, p<0.01), and 76% lower for the Meru (OR = 0.24, 95%CI = 0.12–0.48, p<0.001). Symptoms of diarrhoea and fever were significantly more common in the Maasai compared to the Meru. Maasai children were also more often reported to have recently had pneumonia, with 12% of Maasai children reported to have had pneumonia compared to only 3–4% in all other ethnicities.

Maasai children were not at a disadvantage for all forms of illness. Reported incidence of malaria was significantly more common among the Rangi compared to the Maasai. Furthermore, Meru children, who otherwise appear to be relatively advantaged on most measures, had considerably elevated odds of stating the child had suffered from a worm infection, with 17% of children reported to have had worms in the Meru compared to 5–7% for other ethnic groups. **File S5** also reports ethnic comparisons when carers were asked whether the child had *ever had* each symptom/illness. This analysis produces a similar picture. Notably the odds of having *ever had* malaria are substantially higher for both the Sukuma (OR = 1.58, 95%CI = 0.94–2.65, p<0.1) and Rangi (OR = 2.08, 95%CI = 1.15–3.37, p<0.05) compared to the Maasai. Unexplained between-village variance in child symptoms/illness ranged between 1% (cough/flu) to 14% (pneumonia) of total variance without ethnicity, but reduced substantially after adding ethnicity into each model (**File S5**).

Supplementary analyses confirm that rainfall is a strong correlate of village differences in child health (**File S5**). For example, a child living in a village in the wettest quartile for annual rainfall had both substantially higher HAZ (B = 0.40, 95%CI = 0.09–0.71, p<0.001) and WHZ scores (B = 0.41, 95%CI = 0.19–0.65, p<0.001). Including this effect in our multivariate analyses also reduces the magnitude of ethnic comparisons, particularly when comparing Maasai and Meru children, and particularly for WHZ scores (i.e. acute malnutrition), although most comparisons remain statistically significant. This attenuation suggests that rainfall at least part mediates the ethnic differences reported here. Presence of a health clinic/dispensary and distance from district capital however were not clearly associated with child health, at least with regard to anthropometric indicators (**File S5**).

### Are there ethnic differences in the proximate determinants of child health?

Food insecurity was high across the study villages. Only 9% of households were categorized as “*food secure*”, and 6% as experiencing “*mild food insecurity*”. The majority of households were either categorised as “*moderately food insecure*” (39%); meaning food quality was sacrificed frequently, by eating a monotonous diet or undesirable foods sometimes or often, and/or cutting back on quantity by reducing the size of meals or number of meals, rarely or sometimes; or “*severely food insecure*” (46%); meaning they cut back on meal size or number of meals often, and/or experienced any of the three most severe conditions (running out of food, going to bed hungry, or going a whole day and night without eating) at least once a month [37]. **Table 4** shows the percentage of households in each food insecurity category and the results of logistic regression analyses predicting the odds of being severely food insecure by ethnic group. Maasai were substantially more likely to be categorized as severely food insecure compared to any other ethnic group. At the most extreme comparison, four out of five of Maasai households were classified as “severely food insecure”, compared to only one in four Meru households, representing a 87% reduction in the adjusted odds of severe food insecurity when comparing Meru to Maasai (OR = 0.13, 95%CI = 0.07–0.24, p<0.001). As anticipated food insecurity was significantly higher during hunger season months (OR = 1.41, 95%CI = 1.04–1.92, p<0.05, SI5).

**Figure 3:**
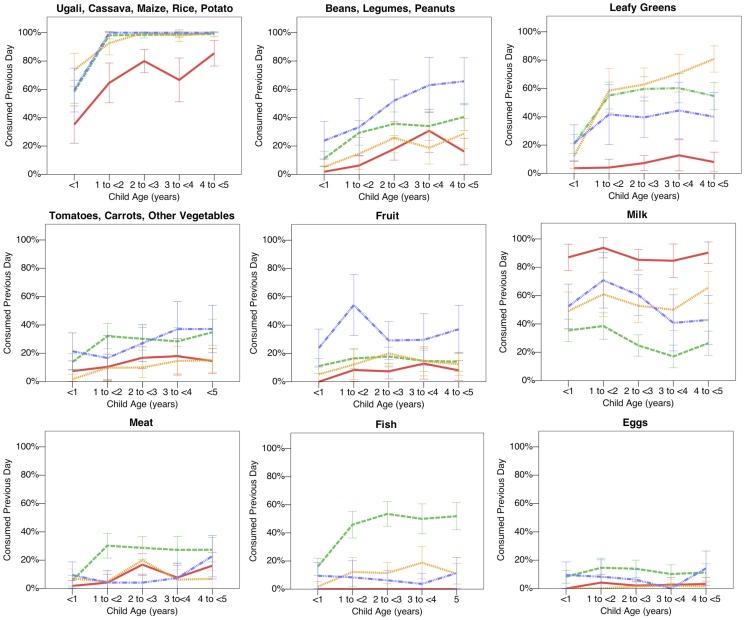
Diet by Age and Ethnicity. Dietary analyses reveal substantial ethnic variation in reported child diet. Red solid line  =  Maasai children; orange dotted line  =  Rangi children; green dashed line  =  Sukuma children; blue dotted and dashed line  =  Meru children. Data plotted only for the 31 villages sampled outside of the hunger season (n = 1903 children), to avoid confounding between ethnicity and season of sampling. 95% confidence intervals calculated using normal approximation. See **File S5** for multilevel logistic regression models predicting food consumption for all children and villages adjusted for child age, child sex, hunger season and a random effect for village.

There are large differences between ethnic groups in the specific food items consumed in the previous day. These differences are illustrated in **Figure3**, which plots ethnic differences in child diet, by food category and child age (see **File S5** and text below for adjusted odds ratios from multivariate analyses including all villages). Consumption of carbohydrate-rich staple foods, such as ugali, was substantially lower among the Maasai compared to all other ethnic groups, where such foods were eaten almost universally by all children between the ages of one and five years. At the largest comparison, the age-adjusted odds of consuming carbohydrate-rich foods was almost six times higher for the Meru compared to the Maasai (OR = 5.92, 95%CI = 2.69–13.05, p<0.001). Maasai children were also the least likely to have eaten beans, legumes or peanuts, and leafy green vegetables compared to other ethnic groups, but there was no significant difference in the odds of consuming tomatoes, carrots or other vegetables. Fruit consumption was significantly lower in the Maasai compared to all other ethnic groups, but particularly compared to the Meru, where around 30–40% of children ate fruit in the previous day, compared to around 5–10% of Maasai children (OR = 2.59, 95%CI = 1.25–4.69, p<0.01).

Meat consumption occurred at low frequency for Maasai, Rangi and Meru children (around 10%). However, the odds of meat consumption was significantly higher for the agro-pastoralist Sukuma (OR = 1.98, 95%CI = 1.09–3.59, p<0.05). Fish consumption was also low for most ethnic groups, but particularly low for Maasai children (2% ate fish on the previous day), and considerably more common in the Sukuma (53% ate fish on the previous day). This difference is likely attributable to the proximity of many Sukuma villages to Lake Victoria (**Figure1**), with ethnic differences attenuated in adjusted analyses including a random intercept for village (**File S5**). Egg consumption was low for all ethnic groups, with borderline significantly lower consumption for Maasai compared to Sukuma and Meru children. Cow's milk was the only food category consumed significantly more often in the Maasai, and was consumed by around 90% of the children in previous day during non-hunger season months, compared to around 30–70% among other ethnic groups (**Figure3**). At the largest comparison, the odds of milk consumption was 77% lower in the Sukuma compared to the Maasai (OR = 0.23, 95% 0.15–0.35, p<0.001).

**Figure 4:**
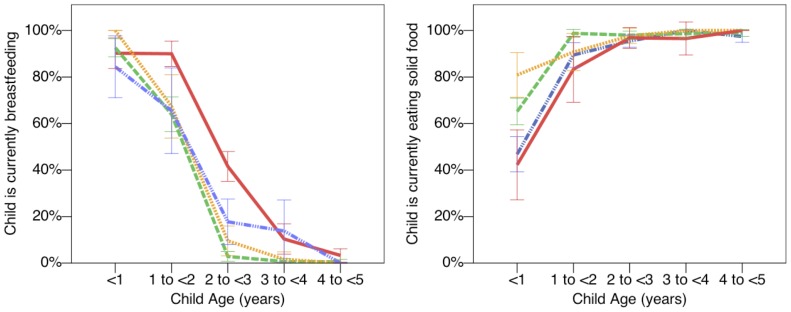
Behaviour by Child Age and Ethnicity. Current patterns indicate relatively delayed weaning in the Maasai compared to other ethnic groups. Red solid line  =  Maasai children; Orange dotted line  =  Rangi children; green dashed line  =  Sukuma children; blue dotted and dashed line  =  Meru children. Data plotted for all 56 villages. 95% confidence intervals calculated using normal approximation.

Breastfeeding behaviour varied by ethnic group (**Table 5**). In all ethnicities, over 90% of children were reported to have been fed colostrum in early infancy, although this was significantly less common among the Sukuma (91% vs. 96% or more for all other ethnic groups). Most notably Maasai children were more likely to be currently breastfeeding compared to children of any other ethnicity, even adjusting for child age. This implies a relatively late age at weaning (see **Figure4**, which plots the percentage of children currently breastfeeding by age and ethnic group). There was also an indication that Maasai and Meru children under the age of one were less likely to be currently eating solids; suggestive of relatively low levels of complementary feeding (**Figure4**). However this difference was not significant in multivariate models (**Table 5**). Unexplained village-level variance in food insecurity, child diet and breastfeeding analyses ranged between 15–49% without ethnicity, but reduced substantially in most cases after adding ethnicity into each model (**File S5**)

**Figure 5:**
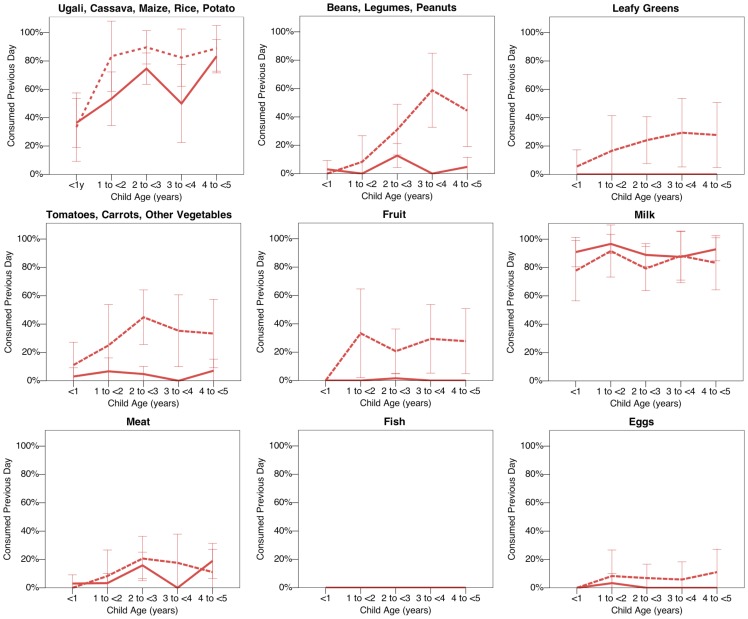
Diet by Age for Pastoralist and Farming Maasai Households. Maasai child diets differ depending on livelihood. Red solid line  =  Maasai pastoralist children; red dashed line  =  Maasai farming children. Data plotted only for the 7 villages containing Maasai households sampled outside of the hunger season (n = 298 children). 95% confidence intervals calculated using normal approximation. See **File S5** for multilevel logistic regression models predicting food consumption for all Maasai children adjusted for child age, child sex, hunger season and a random effect for village.

Vaccination coverage was high for all ethnicities, with over 90% of all children reporting to have had recommended vaccinations for BCG, Polio and DPT (**Table 6**). However, Maasai children were less likely to have received a measles vaccination compared to the Rangi, Meru and “Other Ethnicity” categories. In addition the Meru were more likely to have reported their children had received a Polio vaccination than the Maasai, and the Rangi were more likely to report children had received a DPT vaccination than the Maasai. Reported levels of Vitamin A supplementation varied between 60% among the Sukuma to 89% among the Meru. Vitamin A supplementation was significantly more common in the Meru than in the Maasai (**Table 6**). Between 10–20% of unexplained variance in vaccination and vitamin supplementation was between villages, but this was not notably reduced by the inclusion of ethnicity into each model.

### How do Maasai households that rely on livestock herding compare to those that rely on farming?

We examined associations between primary occupation and child health outcomes among the Maasai adjusting for the same set of covariates, and found that Maasai pastoralists appear to be at a relative disadvantage when compared to Maasai farmers (see the “livelihood” row in Tables 2–6). With regard to anthropometric measurements, adjusted estimates place farmers as 0.39 Z-scores in height-for-age (B = 0.39, 95%CI = 0.06–0.71, p<0.05), and 0.39 Z-scores in terms of weight for age (B = 0.39, 95%CI = 0.13–0.65, p<0.05) above pastoralists. For the most part, reported occurrence of specific illnesses/symptoms did not differ between Maasai farmers and pastoralists. However, Maasai farmers more commonly stated that their children had worms in the past 3 months compared to Maasai pastoralists (OR = 4.91, 95CI% = 1.41–17.04).

Pastoralists were more often categorized as severely food insecure (81%) compared to farmers (72%), however this difference was not statistically significant in adjusted analyses. Dietary analyses confirm a number of differences between Maasai farmer and pastoralist food intake (**Figure 5**; see also **File S5** for adjusted odds ratios). The children of Maasai farmers had a significantly higher odds of eating leafy greens (OR = 3.11, 95%CI = 1.72–5.61, p<0.001) and fruit (OR = 2.95, 95%CI = 1.44–6.02, p<0.001), and were borderline significantly more likely to have recently eaten carbohydrate-rich staple foods (OR = 1.76, 95%CI = 0.99–1.42, p<0.01), tomatoes, carrots or other vegetables (OR = 1.82, 95%CI = 0.97–3.40, p<0.1) and eggs (OR = 2.40, 95%CI = 0.95–6.07, p<0.1). There was no significant difference in the recent consumption of milk, meat or fish between farmers and pastoralists. Multilevel regression models also estimated no overall significant difference in the consumption of beans, legumes and peanuts. However analysing these data by child age indicates more common intake of these foods among the children of farmers above the age of three years (**Figure 5**). Breastfeeding behaviour and vaccination coverage did not differ between the children of Maasai pastoralists and those of Maasai farmers.

## Discussion

### Ethnic Differences in Child Health in Northern Tanzania

We demonstrate the existence of strong ethnic differences in child health. Maasai children are substantially disadvantaged compared to neighbouring ethnic groups and this disadvantage is concentrated in the majority of Maasai who record livestock herding as their primary livelihood. Many authors have emphasised the numerous difficulties faced by the Maasai, and by pastoralist populations more generally, [9]–[13], leading us to the strong expectation that the health of Maasai children would be relatively poor. However, this is the first study to quantitatively contrast child health and its proximate determinants between the Maasai and neighbouring ethnic groups using large sample data. Differences in chronic malnutrition are particularly striking. The most recent Tanzanian DHS (2010) states that 45% of children resident in rural areas are stunted [41], while we place 59% of Maasai pastoralist children, but only 21% of Meru children, in the same category. Self-report data further suggest that the Maasai are at an elevated risk of certain illnesses/symptoms, including pneumonia, which is closely linked to poverty and malnutrition [1], but also fever and diarrhoea. Food insecurity, a well-substantiated cause of poor child health in low-income settings [42], presents a strong candidate proximate determinant behind these differences. Maasai were considerably more likely to be categorised as severely food insecure compared to all other ethnic groups (78% vs. 24–47% respectively). Maasai children also had a lower intake of carbohydrate-rich staple foods, nutritious leafy green vegetables and fruit, and important non-milk sources of protein (beans, legumes and peanuts, fish, eggs). As has been reported for other pastoralist populations in Tanzania [27], the Maasai also had lower vaccination coverage, consistent with relatively low engagement with/availability of health services.

Maasai children are unmistakeably at an overall disadvantage, but by considering a broader range of health measures beyond anthropometric status, our study also indicates that they experience a reduced vulnerability to at least some pathogens. Maasai children had a lower self-reported incidence of malaria than the Sukuma and Rangi. In addition, Maasai households reported the lowest incidence of worm infections, which was markedly more common in otherwise relatively advantaged Meru children. Maasai farmers also reported more worm infections compared to pastoralist households. Previous studies of pastoralist populations have recorded similar patterns; with both Kenyan Turkana and Malian Tuareg reported to have lower levels of intestinal parasites compared to agriculturalists, see [43], [44] cited in [45]: p505. One potential explanation is that the low rainfall environment of the Maasai, particularly in pastoralist villages, reduces opportunities for pathogen transmission. Corbett et al. [46], p.208, for example, suggest that the relative lack of standing water in semi-arid areas occupied by Turkana pastoralists lowers mosquito frequency and consequently malaria exposure compared to neighbouring wetter areas inhabited by farmers.

Maasai children were also more likely to have recently consumed milk than other ethnic groups, consistent with previous research reporting high milk consumption for pastoralists, e.g. [33], [44], [47]. A considerable body of research has emphasised the benefits of milk consumption for child health and growth [48]–[51], and milk consumption has been proposed to account for the relatively tall adult height of east African pastoralist populations, including the Maasai (e.g. [52], [53]). Our study suggests that, at least with regard to early childhood health and growth, potential benefits of higher milk consumption do not offset the challenges of severe food insecurity and a greater burden of illness faced by the Maasai. Milk consumption may however be an important contributing factor to continued growth into and during adolescence; the period of growth responsible for the relatively tall attained adult height for African pastoralists like the Maasai and Turkana [53], [54]. In this context, we caution that population-level comparisons in adult height should not be interpreted as evidence of health disparities. Indeed, adult height is positively correlated with child mortality when comparing African nations [55], a trend that may be partly accounted for by mortality selection, i.e. early death of those children that would otherwise have grown up to be short adults [55], and the evolved adaptation of later growth patterns to related features of the ecology [56].

It is also interesting that, despite the apparent availability of cow's milk, breastfeeding was relatively prolonged in the Maasai compared to neighbouring ethnic groups. This finding is consistent with Sellen and Smay [57] who conclude that weaning food availability is poorly predictive of weaning age, at least when making population-level comparisons. Wander and Mattison [58] also report that cattle-holding households did not wean children earlier than households without cattle in the Chagga ethnic group in the Kilimanjaro region.

A comparison of household characteristics (**Table 1**) and a review of the relevant literature suggest that a combination of factors may be ultimately responsible for marked ethnic differences in child health we observe. Unsurprisingly, socioeconomic variation runs parallel to differences in child health and food insecurity, with Maasai households being relatively poor and undereducated. Furthermore as anticipated, sampled Maasai households were resident in villages with lower annual rainfall, which makes both productive pastoralism and farming more difficult. In contrast, the Meru, who generally had the best child health outcomes, occupy the relatively high rainfall, fertile slopes of mount Meru in close proximity to Arusha city, benefiting from increased health care and education infrastructure, along with opportunities for beneficial forms of livelihood diversification. Supplementary analyses (**File S5**) confirm that annual rainfall is a strong correlate of village-level differences in child health, and contributes to the ethnic differences documented here. Interestingly, however, distance to district capital and presence of a health clinic/dispensary were not predictive of child health, at least with regard to anthropometric indicators. Yet, we caution these measures may be only weakly informative of the quality of services provided. Indeed, many rural health clinics are poorly stocked and understaffed. Previous research has also emphasized the Maasai suffer from relatively ineffective interaction with health services, even when health services are available, due to language differences, distrust and potentially culturally inappropriate services [7], [12].

The objective of the present study is to provide a systematic comparison of ethnic differences in child health across a broad range of measures. Further analysis, guided by the patterns established here, would be instructive in quantifying the relative contribution of alternative explanatory factors to the ethnic differences observed, unpicking the causal chain from distal to more proximate health determinants. Such analyses should include a consideration of notable ethnic differences in household structure (**Table 1**), including a particularly high frequency of female-headed and polygynous households among the Maasai [59], [60], but see [61]. Differences in ratio of dependent children to productive adults may also be important [62]. Furthermore, as has been emphasised in prior literature [28], [63] ethnic differences in child health may be partly attributable to cultural norms more or less independently of ecological, socioeconomic or demographic pathways. For example, the Maasai are notable for practicing dietary restrictions during pregnancy [64], which could have negative consequences for children.

### Limitations

Household sampling was random within villages, but villages were not randomly sampled from the districts and regions of northern Tanzania included in the study site (see *Methods*). With the absence of alternative forms of representative data, we caution against extrapolating observed patterns outside their context of measurement. Nonetheless, given the large geographic spread of villages, and large sample sizes achieved, we believe our findings broadly approximate wider trends, and we are certainly not aware of any comparable datasets of this size enabling ethnic comparisons. All analyses adjust for hunger season and include random-effects for village in attempt to control for potential confounding effects of temporal variation in child health across the two-year study period. Such controls are unavoidably imperfect and so we acknowledge that variation in the timing of household surveys represents a limitation of our analyses. It is also possible that we overestimate the disadvantages of the Maasai that might be observed in a more typical year because our surveys were conducted during and immediately following the 2009 drought, which is believed to have been particularly devastating for Maasai pastoralists [18]. However, droughts are a periodic occurrence, and some of the largest ethnic differences we observe are in height-for-age, a measure reflecting chronic nutritional stress, suggesting our conclusions reflect longer-term trends of Maasai disadvantage.

We recognize that several health measures, including measures of disease incidence, vaccination and diet, rely on self-report, and as such may be subject to recall issues and other forms of respondent bias e.g. familiarity and conceptual recognition of health conditions. For example, there may be much ambiguity in the parental categorisation of child respiratory illness as cough/flu vs. pneumonia (although our analysis indicates these outcomes have a distinct distribution). In the absence of comparable large-sample data from other sources, we believe our use of self-report data is justified for the purpose of this study; establishing important sources of ethnic variation, which through directed further study may inform future interventions aiming to improve child health and reduce inequality. More detailed comparative analysis of ethnic variation in diet, going beyond the crude food group categories and recall method used here, would also be valuable. This would, for example, enable a fuller consideration of the impact of animal sourced foods and milk consumption [51], [65], and the role of “indigenous knowledge” regarding food preparation and use, as emphasized by Oiye et al. [66] in a detailed dietary study of a Kenyan Maasai community.

### Implications and Conclusion

Our conclusions echo wider international findings that marginalised ethnic groups that rely on alternative modes of production to agriculture are disadvantaged in terms of health outcomes and access to health services [6]–[8]. There are a large number of organisations that seek to improve the situation of the Maasai [67]. Our results reinforce support for these initiatives, particularly those combating issues of food insecurity and child nutrition, along with a greater emphasis on the specific needs of pastoralists in Tanzanian health policy [12]. We also emphasize that the situation of the Maasai is perhaps particularly troubling given on-going “land grabbing” conflicts (reviewed in [19]), and the widespread commodification of their culture for profit, both inside and outside of Tanzania. Studies of the impact of cultural tourism attractions, such as so-called “cultural bomas”, where Maasai pose for photographs, sing, dance and sell handmade products to tourists in an artificial settlement, indicate a complex mix of benefits and costs to local communities [16], [17], [68]. Moreover, it has been estimated that many foreign companies made multi-million dollar profits in the last decade from Maasai-themed products, ranging from clothing and bed covers, to car accessories and stationary, capitalising on the exotic image of the Maasai to a western audience [69]. In recognition that these profits rarely find their way to the Maasai people, efforts are being made to enforce intellectual property rights, including the recent formation of a Maasai Intellectual Property Initiative (maasaiip.org).

Our results caution against a simple narrative that numerical minority status alone necessitates disparities in health. The Maasai are one of many ethnic groups in Tanzania that make up only a very small fraction of its total population, and, in the area covered by SFTZ, outnumber the Meru who had the best child health outcomes in this study. Likewise, the Sukuma, by far the most common ethnic group in Tanzania, were not the most advantaged. Indeed the Maasai can only be viewed a marginalised “minority” when we consider broader distinctions of ecology, livelihood, and wider ethnic and cultural distinctions in Tanzania (e.g. Nilotic vs. Bantu). Furthermore the usage of the term “indigenous” in Africa, and its specific and popular application to the Maasai, is not without controversy [70], [71] Maasai-focused organisations have been instrumental in their use of the indigenous label [67], but they cannot be considered “first peoples” in the region, having migrated into Kenya and Tanzania only in last several hundred years, nor are they the only non-agriculturalists in Tanzania. Indeed there exist many other ethnic groups in Tanzania who antedate the Maasai, and/or share similar issues of cultural and economic marginalisation, but are rarely included in the indigenous rights movement [70], [71]. Whatever the case, irrespective of both minority and indigeneity, contrasting welfare indicators between ethnic groups is an important endeavour because it identifies vulnerable communities in need and, through directed further analysis, can improve our understanding of the structural and cultural factors underlying health inequality amendable to change, e.g. [5], [28].

Much has been written about the health consequences pastoralists face when transitioning to agriculture and/or permanent settlement. Previous research has largely relied on small opportunistic samples and produced conflicting conclusions [43], [46], [72]–[74], most likely reflecting differences among sites in agricultural productivity, compatibility with continued pastoralist production and exposure to diseases in more permanent homesteads, and the varying existence of economic alternatives contingent on settlement. In this study, we found that Maasai who identify as farmers experience lower food insecurity (although this difference was not statistically significant), and report their children have less restricted diets (e.g. higher reported consumption of carbohydrate-rich food, fruit and certain vegetables) and better nutritional status as indicated by anthropometric measures. It is tempting to see this as evidence of positive effects of farming on child health, with the implication farming should be encouraged. However, such an interpretation cannot be inferred from the current analysis. Farming households were also resident in higher rainfall villages, and were more often resident in villages with a health clinic or dispensary (**Table 1**). These differences highlight that cultivation is unlikely to be ecologically or economically feasible in all contexts, and that service provision may also vary with livelihood shifts. Consequently cross-sectional analyses may be vulnerable to false comparisons. Previous research has also emphasized that wealthy pastoralists diversify as a risk avoidance strategy, while poorer households may do so out of necessity [73], and our study can not distinguish between these alternatives. Clearly, longitudinal studies are needed to better understand the health consequences of subsistence transitions.

We conclude by emphasizing the value of disaggregated data by ethnicity and livelihood in the measurement of population health. Given the lack of ethnicity data in recent national surveys in Tanzania, there has been unsurprisingly little discussion of the role of ethnicity in structuring inequalities in health and progress towards development targets (e.g. [2], [75]). Previous research has also specifically lamented the lack of data available for monitoring the health of Tanzania's pastoralists and their contribution to social and economic disparities within the nation [12]. Without such data, important dimensions of inequality are likely to go underestimated and unaddressed [7]. Furthermore, as we demonstrate, ethnicity and livelihood type are related not only to child health, but also a range of other ecological, socioeconomic, demographic and cultural variables. We also demonstrate high levels of clustering in child health outcomes and determinants at the village-level. Naïve analysts of aggregate data may therefore easily confound such parameters and draw erroneous conclusions regarding health determinants. We encourage future DHS in Tanzania to reincorporate the collection and dissemination of ethnicity data, and, where such data remain absent, we urge analysts to be vigilant of the limitations to ignoring ethnic and spatial variation (see also [61]). Finally, we hope that this study contributes to an increased recognition of the disadvantages currently experienced by the Maasai, and the support and development of initiatives targeting their specific needs.

## Supporting Information

File S1 **Ethnicity and Sampling by Village, District and Region.**
(PDF)

File S2 **Supporting Information on Household-Level Data.**
(PDF)

File S3 **Supporting Information on Village-Level Data.**
(PDF)

File S4 **Supporting Information on Child-Level Data.**
(PDF)

File S5 **Full Model Output.**
(PDF)
