# Supplementary material for: Ethnicity and Child Health in Northern Tanzania: Maasai Pastoralists Are Disadvantaged Compared to Neighbouring Ethnic Groups
Source: PLoS One. 2014 Oct 29;9(10):e110447. doi: 10.1371/journal.pone.0110447 (PMC4212918; doi:10.1371/journal.pone.0110447)
Supplement: File S1 — Ethnicity and Sampling by Village, District and Region. (PDF) [file pone.0110447.s001.pdf]

Supporting Information 1. Ethnicity and Sampling by Village, District and Region

Table S1 shows the most common ethnic group for each village, along with household and child sampling by village, district and region. Maasai, Sukuma, Meru and Rangi were the most common ethnic groups sampled, collectively accounting for 60.4% of households sampled. A variety of other ethnic groups were also sampled at relatively low frequently making up the ‘Other Ethnicity’ category in our analyses. This includes the Arusha (6.4%), the Wanda (4.7%), the Iraqw (4.3%), the Turu (3.0%), the Mbugwe (2.9%), the Gogo (2.0%) and a large number of ethnic groups each accounting for <2% of the sampled households. The other category also includes 98 households where ethnicity data was not recorded for unknown reasons, 54 of which contributed child health data. Repeating the analyses presented in this paper excluding cases with missing ethnicity data does not meaningfully alter estimated ethnic differences.

Table S1:
Household and Child Sampling by Area and Ethnicity

AREA			TRIBE	SAMPLING		
REGION (n=7)	DISTRICT (n=13)	VILLAGE (n=56)	Most Common Tribe (% of sampled households)	Households sampled (n=3584)	Households providing child health data (n=2268)	Children sampled (n=3586)
ARUSHA				1308	830	1161
	Arumeru			*346*	*181*	*242*
		King'ori	Meru (87%)	75	36	46
		Leguruki	Meru (88%)	77	35	51
		Samaria	Meru (72%)	60	36	46
		Malula	Meru (47%)	60	37	52
		Njoro	Meru (88%)	74	37	47
	Arusha			*202*	*130*	*175*
		Oldonyowasi	Meru (43%)	60	35	47
		Mzimuni	Arusha (51%)	67	33	43
		Lengijave	Arusha (81%)	75	62	85
	Longido			*461*	*347*	*522*
		Kimokouwa	Maasai (97%)	71	51	71
		Eworendeke	Maasai (93%)	75	47	57
		Tingatinga	Maasai (93%)	60	36	48
		Elerai	Maasai (95%)	60	46	65
		Kiserian	Maasai (99%)	75	64	94
		Sinya	Maasai (100%)	60	53	91
		Kitendeni	Maasai (72%)	60	50	96
	Monduli			*299*	*172*	*222*
		Engaruka Juu	Maasai (76%)	75	41	53
		Migombani	Iraqw (18%)	77	30	39
		Selela	Maasai (74%)	74	48	63
		Naitolia	Arusha (59%)	73	53	67
MARA				180	106	202
	Bunda			*180*	*106*	*202*
		Kabasa	Sukuma (67%)	60	39	74
		Nyatwali	Sukuma (42%)	60	29	51
		Serengeti	Sukuma (75%)	60	38	77
MANYARA				688	421	626
	Babati			*392*	*212*	*333*
		Boay	Rangi (31%)	76	39	61
		Gidas	Iraqw (52%)	66	38	63
		Mandi	Iraqw (95%)	59	34	63
		Mwada	Mbugwe (60%)	71	34	50
		Sangaiwe	Mbugwe (63%)	60	31	43
		Vilima Vitatu	Mbugwe (42%)	60	36	53
	Kiteto			*296*	*209*	*293*
		Ngipa	Gogo (35%)	60	33	48
		Matui	Rangi (36%)	61	48	70
		Engusero	Gogo (32%)	60	43	62
		Makame	Maasai (100%)	56	45	62
		Ndedo	Maasai (93%)	59	40	51
SINGIDA				313	178	274
	Iramba			*135*	*67*	*90*
		Nduguti	Iramba (60%)	75	28	38
		Nkinto	Wanda (50%)	60	39	52
	Singida			*178*	*111*	*184*
		Masweya	Turu (39%)	59	38	67
		Mtunduru	Turu (50%)	60	35	56
		Siuyu	Turu (81%)	59	38	61
DODOMA				436	253	380
	Kondoa			*436*	*253*	*380*
		Kelema Kuu	Rangi (80%)	61	41	60
		Filimo	Rangi (92%)	60	31	47
		Mnenia	Rangi (90%)	60	32	49
		Kingale Juu	Rangi (82%)	74	33	48
		Dalai	Rangi (98%)	60	39	60
		Songolo	Rangi (58%)	60	35	50
		Chandama	Rangi (50%)	61	42	66
MWANZA				180	131	248
	Kwimba			*180*	*131*	*248*
		Mwaboma	Sukuma (98%)	60	46	84
		Mwang'alanga	Sukuma (98%)	60	40	69
		Runele	Sukuma (95%)	60	45	95
SHINYANGA				479	349	695
	Maswa			*239*	*162*	*320*
		Hinduki	Sukuma (97%)	60	48	101
		Ipililo	Sukuma (95%)	59	42	77
		Mwang'holo	Sukuma (98%)	60	41	77
		Zebeya	Sukuma (95%)	60	31	65
	Meatu			*240*	*187*	*375*
		Mbushi	Sukuma (85%)	60	51	111
		Iramaba Ndogo	Sukuma (83%)	60	48	104
		Sapa	Sukuma (87%)	60	48	99
		Makao	Sukuma (38%)	60	40	61
