# Supplementary material for: Ethnicity and Child Health in Northern Tanzania: Maasai Pastoralists Are Disadvantaged Compared to Neighbouring Ethnic Groups
Source: PLoS One. 2014 Oct 29;9(10):e110447. doi: 10.1371/journal.pone.0110447 (PMC4212918; doi:10.1371/journal.pone.0110447)
Supplement: File S2 — Supporting Information on Household-Level Data. (PDF) [file pone.0110447.s002.pdf]

Supporting Information 2: Supporting Information on Household-Level Data

2.1 Household Wealth Index

The Household Wealth Index was calculated on the basis of a principle components analysis (PCA). The PCA was applied to a total of 37 dichotomous variables representing ownership of assets and characteristics of assets at the household level. Owning a particular asset or a better asset increases the value of the index by different amounts determinants by the household's score of the first principal component. The index is scaled so that's its minimum is zero, i.e. by construction the poorest household has a score of zero. The following assets were included*: drinking water source, household flooring type, household roofing type, type of toilet, owns land, house, cart, hoe, motorcycle, bicycle, plough, sewing machine, lantern, wheelbarrow, computer, radio, water tank, video, chair, sofa, bed, cupboard, chest, dining set, car, cell phone, solar panel, watch or clock, drum*. Note the index does include land ownership (yes/no) and items relating to farming such as a plough or hoe, but does not include livestock ownership, despite cattle having a clear economic value. Therefore it should be interpreted as a 'non-livestock wealth index'. Based on household surveys we were able to complete a wealth index for 3480/3584 (97.1%) of surveyed households.

2.2 Household Food Insecurity Access Scale

The Household Food Insecurity (Access) Scale (HFIAS) was used to measure food insecurity (Coates, Swindale, & Bilinsky, 2007). It is a brief survey instrument developed by Food and Nutrition Technical Assistance (FANTA) to access whether or not households have experienced problems with accessing food during the last 30 days. The instrument consists of nine occurrence questions and nine frequency questions and results in a continuous measure or categorical measure of food insecurity. This study uses the categorical measure.

Survey respondents are asked if the situation described in each question has occurred in the past four weeks. If the answer is yes, they are they asked with what frequency did this event occur: rarely (1-2 times), sometimes (3 to 10 times), or often (more than 10 times) during the last 30 days. *"In the past 4 weeks… (1) did you worry that your household would not have enough food?; (2) were you or any household member not able to eat the kind of foods you preferred because of a lack of resources; (3) did you or any household member have to eat a limited variety of foods due to a lack of resources?; (4) did you or any household member have to eat some foods that you really did not want to eat because of a lack of resources to obtain other types of food?; (5) did you or any household member have to eat a smaller meal than you felt you needed because there was not enough food?; (6) did you or any household member have to eat fewer meals in a day because there as not enough food? (7) was there ever no food or eat of any kind in your household because of*

lack of resource to get food? (8) - did you or any household member go to sleep at night hungry because there was not enough food? (9) did you or any household member go a whole day and night without eating anything because there was not enough food?"

The categorical HFIAS measure categorizes households into four levels of household food insecurity: (i) food secure, and (ii) mild food insecurity, (iii) moderate food insecurity and (iv) severe food insecurity. Households are categorized as increasingly food insecure as they respond affirmatively to more severe conditions and/or experience those conditions more frequently. A 'food secure' household experiences none of the food insecurity (access) conditions, or just experiences worry, but rarely. A mildly food insecure (access) household worries about not having enough food sometimes or often, and/or is unable to eat preferred foods, and/or eats a more monotonous diet than desired and/or some foods considered undesirable, but only rarely. But it does not cut back on quantity nor experience any of three most severe conditions (running out of food, going to bed hungry, or going a whole day and night without eating). A moderately food insecure household sacrifices quality more frequently, by eating a monotonous diet or undesirable foods sometimes or often, and/or has started to cut back on quantity by reducing the size of meals or number of meals, rarely or sometimes. But it does not experience any of the three most severe conditions. A severely food insecure household has graduated to cutting back on meal size or number of meals often, and/or experiences any of the three most severe conditions (running out of food, going to bed hungry, or going a whole day and night without eating), even as infrequently as rarely. In other words, any household that experiences one of these three conditions even once in the last four weeks (30 days) is considered severely food insecure.

Reference: Coates J, Swindale A & Bilinsky P (2007) Household Food Insecurity Access Scale (HFIAS) for Measurement of Food Access: Indicator Guide (v.3). Washington DC: Food and Nutrition Technical Assistance Project, Academy for Educational Development, August 2007.

2.3 Missing Data

There is a small amount of missing data for several of the household-level variables summarised in Table 1, due to survey respondents being unwilling or unable to give responses. Out of the 3584 surveyed households, occupation data was missing for 7 cases, highest education level attained was missing for 53 cases, household type (i.e. polygynous/monogamous) missing for 66 cases, religion missing for 9 cases. There were also 60 cases were households provided insufficient data to calculate the household food insecurity access scale (Table 4).
