# Supplementary material for: Ethnicity and Child Health in Northern Tanzania: Maasai Pastoralists Are Disadvantaged Compared to Neighbouring Ethnic Groups
Source: PLoS One. 2014 Oct 29;9(10):e110447. doi: 10.1371/journal.pone.0110447 (PMC4212918; doi:10.1371/journal.pone.0110447)
Supplement: File S3 — Supporting Information on Village-Level Data. (PDF) [file pone.0110447.s003.pdf]

Supporting Information 3: Supporting Information on Village-Level Data

3.1 Rainfall

Mean monthly rainfall data for each village was derived from the 'WorldClim' climatic data resource as the mean monthly total precipitation over the period covering 1950-2000 at a resolution of 1km2 (Hijmans et al. 2005) mapped to a central point of each village.

Hijmans, R.J., S.E. Cameron, J.L. Parra, P.G. Jones and A. Jarvis, 2005. Very high resolution interpolated climate surfaces for global land areas. International Journal of Climatology 25: 1965-1978.

3.2 Distance to District Capital

Distance to District capital was calculated for each village using the straight-line distance between the mean coordinates of all sampled households within each village and the central point of the District capital in km.

3.3 Health Dispensaries/Clinics

In each village, village leaders were interviewed about the availability of key services. This information was used to code the presence/absence of health dispensaries or clinics either inside a village or within easy walking distance. Note that this variable is based purely on the presence or absence of a health service and does not take into account the quality of service provision.

3.4 Hunger Season

Villages were sampled over a two-year period (July 2009 to May 2011) and across an extensive geographic area which straddles two climatic zones experiencing either bimodal or unimodal rainy seasons, influencing the timing of so-called "hunger" or "lean" seasons. Although annual rainfall patterns are erratic, the hunger season generally occurs from October to December in the bimodal zone; while in the unimodal zone, an overlapping but longer hunger season generally falls from November to February (see the USAID Famine Early Warning System http://www.fews.net/east-africa/tanzania/seasonal-calendar/tue-2013-12-17). Based on this monthly categorization we coded whether or not each village was sampled during in the hunger season, with all villages from the Regions of Arusha, Mara and Mwanza considered to be in the bimodal zone, and the Regions of Shinyanga, Dodoma, Manyara and Singida in the unimodal zone. A binary coding of "not hunger season" (31 villages) vs. "hunger season" (25 villages) was included in all models.
