# Supplementary material for: Ethnicity and Child Health in Northern Tanzania: Maasai Pastoralists Are Disadvantaged Compared to Neighbouring Ethnic Groups
Source: PLoS One. 2014 Oct 29;9(10):e110447. doi: 10.1371/journal.pone.0110447 (PMC4212918; doi:10.1371/journal.pone.0110447)
Supplement: File S4 — Supporting Information on Child-Level Data. (PDF) [file pone.0110447.s004.pdf]

Supporting Information 4: Supporting Information on Child-Level Data

4.1 Age of Child at Sampling

Figure S1 shows that while there is clumping around exact ages (e.g. 2 years, or 2 years 6 months) as is commonly found with self-reported age data, the study sampled children across the full age range of 0-60 months at a roughly even distribution.

Figure S1: Histogram of Age of Child in Months at Anthropometric Survey

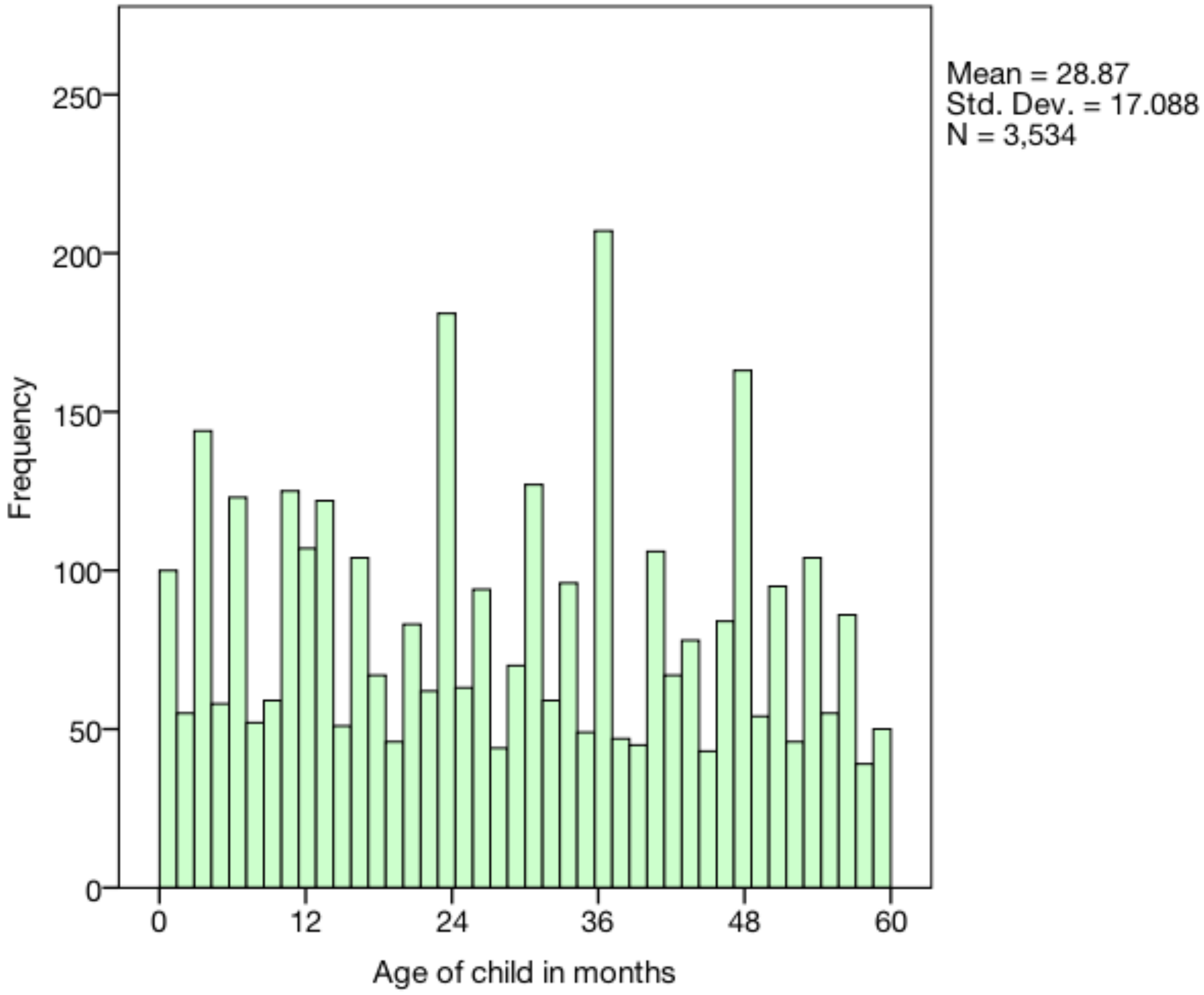

4.2 Calculation of HAZ, WHZ and WAZ Scores

HAZ, WHZ and WAZ scores derived in IBM SPSS v.20 using WHO supplied syntax, which automatically removes extreme cases likely to have resulted from measurement error i.e. incorrectly recorded child age, height or weight. For WAZ scores of <-6 or >5 are removed, for HAZ scores of <-6 or >6 are removed, and for WHZ scores of <-5 or >5 are removed.

http://www.who.int/childgrowth/software/en/

4.3 Foods included in the Diet Survey

Table S2 provides descriptive data on the various foods included on the child diet survey. Child carers were asked whether or not each food stuff had been consumed in the day prior to the survey. These foods were then divided into nine categories. No information was gathered with regard to the volume of food consumed or its quality.

Table S2: Percentage of children reported to consume each food included on the diet survey
Response to Survey Question: "Has the child eaten X in the previous day?"

Food	Yes (%)	Food	Yes (%)
1. Carbohydrate Rich Food		5. Fruit	
Ugali (maize meal)	67	Banana	9
Muhogo/Cassava	4	Papaya	6
Maize	25	Pineapple	3
Rice	17	Orange	8
Potato	14	Other fruit	5
Other carbohydrates	26	6. Milk	48
2. Beans, Legumes, Peanuts		7. Meat	
Beans/Legumes	19	Chicken	7
Peanuts	11	Goat	8
3. Leafy Greens	44	Cow	9
4. Tomatoes, Carrots, Other Vegetables		Bushmeat	1
Tomatoes	15	Other Animal	4
Carrot	4	8. Fish	18
Other vegetables	4	9. Eggs	7

4.4 Missing data on child-level measures

There is a small amount of missing data for several of the child-level variables used as dependent variables in this analysis, due to survey respondents being unwilling or unable to give responses. Out of the 3586 children, subjective health ("good vs./ frequently ill") was missing for 1 case (see Table 3). For 391 out of 3586 children the household head did not know if the child was currently breastfeeding (se Table 5).

In addition carers where only asked if a child had a specific illness/symptom over the past 3 months, as opposed to over "ever" for 36/56 villages.
