# Supplementary material for: Ethnicity and Child Health in Northern Tanzania: Maasai Pastoralists Are Disadvantaged Compared to Neighbouring Ethnic Groups
Source: PLoS One. 2014 Oct 29;9(10):e110447. doi: 10.1371/journal.pone.0110447 (PMC4212918; doi:10.1371/journal.pone.0110447)
Supplement: File S5 — Full Model Output. (PDF) [file pone.0110447.s005.pdf]

## Supporting Information 5: Full Model Output

Tables S3-S9 show the full model output for the fixed and random effects for each multilevel regression model corresponding to Tables 2-6 in the main text. Note that Variance Partition Components (VPC) for within and between village variances are calculated as the percentage of total variance.

**Table S3a**
**Multilevel Linear Regressions Predicting Child Anthropometric Status**

| | | Height for Age Z Score (n = 3411) | Weight for Height Z Score (n = 3426 ) | Weight for Age Z Score (n = 3507) |
|---|---|---|---|---|
| Fixed Effects | | B coefficient *(95% CIs)* | B coefficient *(95% CIs)* | B coefficient *(95% CIs)* |
| Ethnicity | Maasai | 0.00 | 0.00 | 0.00 |
| | Sukuma | 0.59*** *(0.36 – 0.82)* | 0.44*** *(0.26 – 0.63)* | 0.72*** *(0.55 – 0.89)* |
| | Rangi | 0.22 *(-0.05 – 0.49)* | 0.04 *(-0.18 – 0.27)* | 0.27* *(0.06 – 0.47)* |
| | Meru | 0.92*** *(0.60 – 1.25)* | 0.43** *(0.16 – 0.70)* | 0.87*** *(0.62 – 1.12)* |
| | Other | 0.32** *(0.13 – 0.51)* | 0.26** *(0.10 – 0.41)* | 0.46*** *(0.31 – 0.60)* |
| Livelihood [a] (Maasai only) | Livestock | 0.00 | 0.00 | 0.00 |
| | Farmer | 0.39* *(0.06 – 0.71)* | 0.20 *(-0.09 – 0.48)* | 0.39** *(0.13 – 0.65)* |
| Age (months) | | -0.08*** *(-0.09 – -0.07)* | -0.03*** *(-0.04 – -0.02)* | -0.06*** *(-0.07 – -0.05)* |
| Age (months) squared | | 0.00*** *(0.00 – 0.00)* | 0.00* *(0.00 – 0.00)* | 0.00*** *(0.00 – 0.00)* |
| Child Sex | Male | 0.00 | 0.00 | 0.00 |
| | Female | 0.11* *(0.02 – 0.21)* | 0.05 *(-0.04 – 0.13)* | 0.09* *(0.02 – 0.17)* |
| Hunger Season | No | 0.00 | 0.00 | 0.00 |
| | Yes | -0.21* *(-0.39 – -0.03)* | 0.01 *(-0.14 – 0.15)* | -0.14* *(-0.28 – 0.00)* |
| Constant | | -1.03*** *(-1.27 - -0.78)* | 0.57*** *(0.37 – 0.77)* | -0.24* *(-0.43 – -0.05)* |
| Random Effects *(VPC)* | | | | |
| Village Variance Without Ethnicity | | 0.17 *(4%)* | 0.09 *(5%)* | 0.15 *(10%)* |
| Village Variance With Ethnicity | | 0.08 *(3%)* | 0.04 *(2%)* | 0.04 *(3%)* |
| Child Variance Without Ethnicity | | 2.11 *(93%)* | 1.57 *(95%)* | 1.32 *(90%)* |
| Child Variance With Ethnicity | | 2.11 *(96%)* | 1.58 *(98%)* | 1.35 *(97%)* |

+ p<0.1, * p<0.05, ** p<0.01, *** p<0.001
[a] Livelihood parameters are derived from separate models including only Maasai households.

Table S3b

Multilevel Linear Regressions Predicting Child Anthropometric Status Including Village Rainfall, Distance to District Capital and Presence of Health Services

| | | Height for Age Z Score (n = 3411) | Weight for Height Z Score (n = 3426 ) | Weight for Age Z Score (n = 3507) |
|---|---|---|---|---|
| Fixed Effects | | B coefficient *(95% CIs)* | B coefficient *(95% CIs)* | B coefficient *(95% CIs)* |
| Ethnicity | Maasai | 0.00 | 0.00 | 0.00 |
| | Sukuma | **0.40**** *(0.12 – 0.67)* | **0.28*** *(0.06 – 0.49)* | **0.48***** *(0.28 – 0.69)* |
| | Rangi | 0.17 *(-0.11 – 0.45)* | 0.05 *(-0.18 – 0.27)* | **0.20+** *(0.01 – 0.41)* |
| | Meru | **0.66***** *(0.30 – 1.03)* | 0.24 *(-0.05 – 0.54)* | **0.60***** *(0.32 – 0.87)* |
| | Other | **0.21**** *(0.00 – 0.43)* | **0.18*** *(0.00 – 0.35)* | **0.32***** *(0.16 – 0.48)* |
| Livelihood [a] (Maasai only) | Livestock | 0.00 | 0.00 | 0.00 |
| | Farmer | **0.51**** *(0.16 – 0.86)* | -0.05 *(-0.35 – 0.26)* | **0.31*** *(0.03 – 0.58)* |
| Age (months) | | **-0.08***** *(-0.09 – -0.07)* | **-0.03***** *(-0.04 – -0.02)* | **-0.06***** *(-0.07 – -0.05)* |
| Age (months) squared | | **0.00***** *(0.00 – 0.00)* | **0.00*** *(0.00 – 0.00)* | **0.00***** *(0.00 – 0.00)* |
| Child Sex | Male | 0.00 | 0.00 | 0.00 |
| | Female | **0.11*** *(0.01 – 0.21)* | 0.04 *(-0.04 – 0.13)* | **0.09*** *(0.01 – 0.17)* |
| Hunger Season | No | 0.00 | 0.00 | 0.00 |
| | Yes | **-0.16+** *(-0.34 – -0.02)* | 0.03 *(-0.10 – 0.17)* | **-0.10** *(-0.23 – -0.04)* |
| Village Annual Rainfall | Driest Quartile | 0.00 | 0.00 | 0.00 |
| | Dry Quartile | 0.19 *(-0.08 – 0.47)* | **0.21*** *(0.00 – 0.42)* | **0.29**** *(0.08 – 0.49)* |
| | Wet Quartile | 0.19 *(-0.09 – 0.47)* | **0.28**** *(0.07 – 0.49)* | **0.30**** *(0.10 – 0.50)* |
| | Wettest Quartile | **0.40*** *(0.09 – 0.71)* | **0.41**** *(0.19 – 0.65)* | **0.52***** *(0.29 – 0.74)* |
| Distance to District Capital | Closest Quartile | 0.00 | 0.00 | 0.00 |
| | Close Quartile | **-0.24*** *(-0.50 – 0.01)* | -0.05 *(-0.24 – 0.13)* | -0.18 *(-0.36 – 0.00)* |
| | Far Quartile | 0.03 *(-0.22 – 0.28)* | -0.00 *(-0.19 – 0.18)* | 0.02 *(-0.16 – 0.20)* |
| | Furthest Quartile | -0.17 *(-0.41 – 0.08)* | **0.17+** *(-0.01 – 0.35)* | 0.01 *(-0.17 – 0.19)* |
| Health Clinic/Dispensary | No | 0.00 | 0.00 | 0.00 |
| | Yes | 0.14 *(-0.04 – 0.32)* | -0.03 *(-0.16 – 0.10)* | -0.01 *(-0.12 – 0.14)* |
| Constant | | **-1.13***** *(-1.46 - -0.80)* | **0.39**** *(0.14 – 0.63)* | **-0.38**** *(-0.62 – -0.14)* |
| Random Effects *(VPC)* | | | | |
| Village Variance | | 0.06 *(3%)* | 0.03 *(2%)* | 0.03 *(2%)* |
| Child Variance | | 2.11 *(97%)* | 1.57 *(98%)* | 1.33 *(97%)* |

+ p<0.1, * p<0.05, ** p<0.01, *** p<0.001

[a] Livelihood parameters are derived from separate models including only Maasai households.

**Table S4**
**Multilevel Logistic Regressions Predicting Subjective Health and Self-Reported Incidence of Specific Illnesses/Symptoms**

| | | Subjective Health (n=3585) | *"Has the Child Had X in the Past Three Months?" (n = 2496)* | | | | | |
|---|---|---|---|---|---|---|---|---|
| Fixed Effects | | 'Good' vs. 'Frequently Sick' | Fever | Diarrhoea | Pneumonia | Cough/Flu | Malaria | Worms |
| | | Adjusted Odds Ratio (95% CI) | Adjusted Odds Ratio (95% CI) | Adjusted Odds Ratio (95% CI) | Adjusted Odds Ratio (95% CI) | Adjusted Odds Ratio (95% CI) | Adjusted Odds Ratio (95% CI) | Adjusted Odds Ratio (95% CI) |
| Ethnicity | Maasai | 1.00 | 1.00 | 1.00 | 1.00 | 1.00 | 1.00 | 1.00 |
| | Sukuma | 0.58**<br>*(0.40 – 0.84)* | 1.12<br>*(0.82 – 1.52)* | 1.00<br>*(0.67 – 1.50)* | 0.48<br>*(0.18 – 1.28)* | 1.32<br>*(0.94 – 1.87)* | 1.26<br>*(0.79 – 2.03)* | 1.37<br>*(0.62 – 3.01)* |
| | Rangi | 0.90<br>*(0.58 – 1.38)* | 0.70<br>*(0.48 – 1.03)* | 0.93<br>*(0.56 – 1.52)* | 0.31*<br>*(0.10 – 0.96)* | 1.40<br>*(0.92 – 2.11)* | 1.77*<br>*(1.03 – 3.07)* | 1.05<br>*(0.41 – 2.71)* |
| | Meru | 0.24***<br>*(0.12 – 0.48)* | 0.48**<br>*(0.30 – 0.77)* | 0.28**<br>*(0.13 – 0.60)* | 0.37<br>*(0.10 – 1.39)* | 1.17<br>*(0.71 – 1.93)* | 1.06<br>*(0.54 – 2.05)* | 3.27*<br>*(1.24 – 8.6)* |
| | Other | 0.78<br>*(0.57 – 1.06)* | 0.70*<br>*(0.51 – 0.94)* | 0.76<br>*(0.51 – 1.14)* | 0.43+<br>*(0.18 – 1.02)* | 1.05<br>*(0.75 – 1.45)* | 1.43<br>*(0.91 – 2.27)* | 1.24<br>*(0.57 – 2.69)* |
| Livelihood [a] (Maasai only) | Livestock | 1.00 | 1.00 | 1.00 | 1.00 | 1.00 | 1.00 | 1.00 |
| | Farmer | 0.71<br>*(0.44 – 1.15)* | 1.74*<br>*(1.04 – 2.91)* | 0.63<br>*(0.36 – 1.12)* | 1.00<br>*(0.45 – 2.23)* | 1.20<br>*(1.45 – 3.21)* | 1.51<br>*(0.78 – 2.89)* | 4.91*<br>*(1.41 – 17.04)* |
| Age (months) | | 1.01<br>*(0.99 – 1.03)* | 1.02**<br>*(1.01 – 1.05)* | 1.02<br>*(0.99 – 1.04)* | 1.02<br>*(0.97 – 1.07)* | 1.02*<br>*(1.01 – 1.04)* | 1.05***<br>*(1.03 – 1.07)* | 1.08**<br>*(1.03 – 1.12)* |
| Age (months) squared | | 1.00<br>*(1.00 – 1.00)* | 1.00**<br>*(0.99 – 1.00)* | 1.00***<br>*(0.74 – 1.53)* | 1.00<br>*(1.00 – 1.00)* | 1.00**<br>*(1.00 – 1.00)* | 1.00***<br>*(1.00 – 1.00)* | 1.00**<br>*(1.00 – 1.00)* |
| Child Sex | Male | 1.00 | 1.00 | 1.00 | 1.00 | 1.00 | 1.00 | 1.00 |
| | Female | 0.99<br>*(0.82 – 1.18)* | 1.05<br>*(0.90 – 1.24)* | 0.88<br>*(0.74 – 1.06)* | 1.26<br>*(0.86 – 1.84)* | 1.12<br>*(0.95 – 1.31)* | 1.06<br>*(0.88 – 1.28)* | 1.21<br>*(0.88 – 1.67)* |
| Hunger Season | No | 1.00 | 1.00 | 1.00 | 1.00 | 1.00 | 1.00 | 1.00 |
| | Yes | 1.03<br>*(0.78 – 1.37)* | 1.05<br>*(0.85 – 1.30)* | 1.28<br>*(0.95 – 1.72)* | 0.94<br>*(0.53 – 1.64)* | 0.68*<br>*(0.53 – 0.86)* | 0.79<br>*(0.57 – 1.09)* | 0.82<br>*(0.47 – 1.43)* |
| Constant | | 0.23***<br>*(0.15 – 0.35)* | 0.82<br>*(0.57 – 1.18)* | 0.59*<br>*(0.37 – 0.92)* | 0.05***<br>*(0.02 – 0.17)* | 0.99<br>*(0.67 -1.46)* | 0.14***<br>*(0.08 – 0.23)* | 0.02***<br>*(0.01 – 0.04)* |
| Random Effects *(VPC)* | | | | | | | | |
| Village Variance Without Ethnicity | | 0.22 *(6%)* | 0.09 *(3%)* | 0.15 *(4%)* | 0.52 *(14%)* | 0.04 *(1%)* | 0.15 *(4%)* | 0.39 *(11%)* |
| Village Variance With Ethnicity | | 0.15 *(4%)* | 0.02 *(1%)* | 0.08 *(2%)* | 0.19 *(5%)* | 0.04 *(1%)* | 0.10 *(3%)* | 0.28 *(8%)* |

+ p<0.1, * p<0.05, ** p<0.01, *** p<0.001
[a] Livelihood parameters are derived from separate models including only Maasai households.

Table S5
Multilevel Logistic Regression Predicting Household Food Insecurity (n = 2208)

| | | Household is Severely Food Insecure |
|---|---|---|
| Fixed Effects | | Adjusted Odds Ratio (95% CI) |
| Ethnicity | Maasai | 1.00 |
| | Sukuma | **0.18***<br>*(0.12 – 0.28)* |
| | Rangi | **0.32***<br>*(0.20 – 0.51)* |
| | Meru | **0.13***<br>*(0.07 – 0.24)* |
| | Other | **0.20***<br>*(0.14 – 0.29)* |
| Livelihood [a] (Maasai only) | Livestock | 1.00 |
| | Farmer | 0.78<br>*(0.41 – 1.49)* |
| Age (months) | | 0.99<br>*(0.97 – 1.01)* |
| Age (months) squared | | 1.00<br>*(1.00 – 1.00)* |
| Child Sex | Male | 1.00 |
| | Female | 1.01<br>*(0.83 – 1.21)* |
| Hunger Season | No | 1.00 |
| | Yes | **1.41***<br>*(1.04 – 1.92)* |
| Constant | | **2.44***<br>*(1.57 – 3.79)* |
| Random Effects *(VPC)* | | |
| Village Variance Without Ethnicity | | 0.83 (20%) |
| Village Variance With Ethnicity | | 0.19 (5%) |

+ p<0.1, * p<0.05, ** p<0.01, *** p<0.001
[a] Livelihood parameters are derived from separate models including only Maasai households.

Table S6
Multilevel Logistic Regressions Predicting Child Consumption of Food Categories in Previous Day (n = 3586)

| | | Carbohydrates | Beans, Legumes, Peanuts | Leafy Greens | Tomatoes, Carrots, Other Vegetables | Fruit | Milk | Meat | Fish | Eggs |
|---|---|---|---|---|---|---|---|---|---|---|
| Fixed Effects | | Adjusted Odds Ratio (95% CI) | Adjusted Odds Ratio (95% CI) | Adjusted Odds Ratio (95% CI) | Adjusted Odds Ratio (95% CI) | Adjusted Odds Ratio (95% CI) | Adjusted Odds Ratio (95% CI) | Adjusted Odds Ratio (95% CI) | Adjusted Odds Ratio (95% CI) | Adjusted Odds Ratio (95% CI) |
| Ethnicity | Maasai | 1.00 | 1.00 | 1.00 | 1.00 | 1.00 | 1.00 | 1.00 | 1.00 | 1.00 |
| | Sukuma | **2.97******<br>*(1.65 – 5.36)* | **2.61******<br>*(1.64 – 4.18)* | **4.24******<br>*(2.66 – 6.81)* | 1.43<br>*(0.82 – 2.51)* | 1.19<br>*(0.64 – 2.21)* | **0.23******<br>*(0.15 – 0.35)* | **1.98***<br>*(1.09 – 3.59)* | **5.48****<br>*(2.10 – 14.32)* | **1.93+**<br>*(0.93 – 4.00)* |
| | Rangi | **5.92******<br>*(2.69 – 13.05)* | **1.82***<br>*(1.06 – 3.11)* | **4.18******<br>*(2.52 – 6.92)* | 0.78<br>*(0.40 – 1.51)* | **2.42****<br>*(1.25 – 4.69)* | **0.44******<br>*(0.28 – 0.67)* | 1.34<br>*(0.72 – 2.50)* | **6.22******<br>*(2.28 – 16.97)* | 0.49<br>*(0.15 – 1.59)* |
| | Meru | **4.98****<br>*(2.01 – 12.34)* | **3.87******<br>*(2.12 – 7.02)* | **3.00******<br>*(1.61 – 5.58)* | 1.71<br>*(0.86 – 3.40)* | **2.59****<br>*(1.31 – 5.13)* | **0.51***<br>*(0.30 – 0.87)* | 1.48<br>*(0.71 – 3.13)* | **7.84****<br>*(2.42 – 25.36)* | **2.32+**<br>*(0.97 – 5.56)* |
| | Other | **2.96******<br>*(1.79 – 4.88)* | **1.92******<br>*(1.33 – 2.76)* | **3.78******<br>*(2.61 – 5.48)* | 1.09<br>*(1.06 – 1.12)* | **1.75***<br>*(1.11 – 2.76)* | **0.45******<br>*(0.33 – 0.62)* | **1.91****<br>*(1.21 – 3.02)* | **7.06******<br>*(3.01 – 16.57)* | 1.49<br>*(0.83 – 2.70)* |
| Livelihood [a] (Maasai only) | Livestock | 1.00 | 1.00 | 1.00 | 1.00 | 1.00 | 1.00 | 1.00 | 1.00 | 1.00 |
| | Farmer | **1.76+**<br>*(0.99 – 3.13)* | 1.23<br>*(0.68 – 2.21)* | **3.11******<br>*(1.72 – 5.61)* | **1.82+**<br>*(0.97 – 3.40)* | **2.95****<br>*(1.44 – 6.02)* | 0.69<br>*(0.43 – 1.11)* | 1.46<br>*(0.82 – 2.59)* | 0.83<br>*(0.18 – 3.85)* | **2.40+**<br>*(0.95 – 6.07)* |
| Age (months) | | **1.38******<br>*(1.34 – 1.42)* | **1.12******<br>*(1.09 – 1.14)* | **1.16******<br>*(1.14 – 1.18)* | **1.09******<br>*(1.07 – 1.12)* | **1.09******<br>*(1.06 – 1.11)* | 0.99<br>*(0.97 – 1.00)* | **1.14******<br>*(1.11 – 1.17)* | **1.13******<br>*(1.10 – 1.16)* | **1.05****<br>*(1.02 – 1.08)* |
| Age (months) squared | | **1.00******<br>*(1.00 – 1.00)* | **1.00******<br>*(1.00 – 1.00)* | **1.00******<br>*(1.00 – 1.00)* | **1.00******<br>*(1.00 – 1.00)* | **1.00******<br>*(1.00 – 1.00)* | 1.00<br>*(1.00 – 1.00)* | **1.00******<br>*(1.00 – 1.00)* | **1.00******<br>*(1.00 – 1.00)* | **1.00****<br>*(1.00 – 1.00)* |
| Child Sex | Male | 1.00 | 1.00 | 1.00 | 1.00 | 1.00 | 1.00 | 1.00 | 1.00 | 1.00 |
| | Female | 0.91<br>*(0.71 – 1.18)* | 0.86<br>*(0.72 – 1.01)* | 0.92<br>*(0.79 – 1.07)* | 1.06<br>*(0.88 – 1.28)* | 1.11<br>*(0.91 – 1.35)* | 1.07<br>*(0.92 – 1.23)* | 1.08<br>*(0.91 – 1.28)* | 1.05<br>*(0.85 – 1.29)* | 1.03<br>*(0.78 – 1.36)* |
| Hunger Season | No | 1.00 | 1.00 | 1.00 | 1.00 | 1.00 | 1.00 | 1.00 | 1.00 | 1.00 |
| | Yes | 1.07<br>*(0.66 – 1.72)* | 1.02<br>*(0.68 – 1.53)* | 1.42<br>*(0.90 – 2.23)* | 0.83<br>*(0.47 – 1.47)* | 1.06<br>*(0.56 – 2.01)* | **0.47******<br>*(0.33 – 0.67)* | 1.23<br>*(0.69 – 2.20)* | 1.16<br>*(0.47 – 2.84)* | 1.52<br>*(0.80 – 2.88)* |
| Constant | | **0.07******<br>*(0.04 – 0.13)* | **0.03******<br>*(0.16 – 0.47)* | **0.02******<br>*(0.01 – 0.37)* | **0.03******<br>*(0.02 – 0.07)* | **0.03******<br>*(0.02 – 0.06)* | **4.64******<br>*(3.06 – 7.04)* | **0.02******<br>*(0.01 – 0.04)* | **0.00******<br>*(0.00 – 0.01)* | **0.02******<br>*(0.01 – 0.04)* |
| Random Effects *(VPC)* | | | | | | | | | | |
| Village Variance Without Ethnicity 0.83 (20%) | | 0.96 (23%) | 0.59 (15%) | 1.07 (25%) | 1.04 (24%) | 1.42 (30%) | 0.71 (18%) | 1.31 (28%) | 3.18 (49%) | 1.32 (29%) |
| Village Variance With Ethnicity 0.19 (5%) | | 0.51 (13%) | 0.46 (12%) | 0.62 (16%) | 0.95 (22%) | 1.23 (27%) | 0.34 (9%) | 1.05 (24%) | 2.42 (42%) | 0.96 (23%) |

+ p<0.1, * p<0.05, ** p<0.01, *** p<0.001
[a] Livelihood parameters are derived from separate models including only Maasai households.

Table S7
Multilevel Logistic Regressions Predicting Breastfeeding Behaviour

| | | Child Had Colostrums (n = 3530) | Currently Breastfeeding (n = 3195) | Currently Eating Solids (n = 3586) |
|---|---|---|---|---|
| Fixed Effects | | Adjusted Odds Ratio (95% CI) | Adjusted Odds Ratio (95% CI) | Adjusted Odds Ratio (95% CI) |
| Ethnicity | Maasai | 1.00 | 1.00 | 1.00 |
| | Sukuma | **0.22***** *(0.10 – 0.48)* | **0.15***** *(0.09 – 0.25)* | 1.76 *(0.81 – 3.85)* |
| | Rangi | 0.73 *(0.27 – 1.99)* | **0.27***** *(0.14 – 0.51)* | 2.07 *(0.78 – 5.47)* |
| | Meru | 2.53 *(0.49 – 13.11)* | **0.41*** *(0.20 – 0.84)* | 0.77 *(0.26 – 2.25)* |
| | Other | 0.58 *(0.28 – 1.22)* | **0.27***** *(0.18 – 0.41)* | 1.45 *(0.78 – 2.69)* |
| Livelihood [a] (Maasai only) | Livestock | 1.00 | 1.00 | 1.00 |
| | Farmer | 0.50 *(0.14 – 1.70)* | 0.77 *(0.46 – 1.30)* | 1.43 *(0.70 – 2.92)* |
| Age (months) | | 0.99 *(0.95 – 1.03)* | **0.73***** *(0.69 – 0.77)* | **1.45***** *(1.38 – 1.50)* |
| Age (months) squared | | 1.00 *(1.00 – 1.00)* | **1.00***** *(1.00 – 1.00)* | **1.00***** *(0.86 – 1.00)* |
| Child Sex | Male | 1.00 | 1.00 | 1.00 |
| | Female | 1.12 *(0.81 – 1.54)* | 1.01 *(0.79 – 1.29)* | 1.14 *(0.86 – 1.54)* |
| Hunger Season | No | 1.00 | 1.00 | 1.00 |
| | Yes | 1.38 *(0.77 – 2.47)* | **1.42+** *(0.97 – 2.08)* | **0.36**** *(0.18 – 0.70)* |
| Constant | | 49.19 *(21.04 – 115.01)* | **928.61***** *(396.68 – 2173.89)* | **0.19***** *(0.09 – 0.40)* |
| | | | | |
| Village Variance Without Ethnicity | | 0.95 (22%) | 0.71 (18%) | 1.46 (31%) |
| Village Variance With Ethnicity | | 0.48 (13%) | 0.26 (7%) | 1.17 (26%) |

+ p<0.1, * p<0.05, ** p<0.01, *** p<0.001
[a] Livelihood parameters are derived from separate models including only Maasai households.

Table S8
Multilevel Logistic Regressions Predicting Vaccination Coverage and Vitamin A Supplementation (n = 3586)

| | | BCG Vaccination | Polio Vaccination | DPT Vaccination | Measles Vaccination | Vitamin A Supplementation |
|---|---|---|---|---|---|---|
| Fixed Effects | | Adjusted Odds Ratio (95% CI) | Adjusted Odds Ratio (95% CI) | Adjusted Odds Ratio (95% CI) | Adjusted Odds Ratio (95% CI) | Adjusted Odds Ratio (95% CI) |
| Ethnicity | Maasai | 1.00 | 1.00 | 1.00 | 1.00 | 1.00 |
| | Sukuma | 1.38 *(0.64 -2.99)* | 1.49 *(0.73 – 3.03)* | 1.34 *(0.70 – 2.62)* | 0.84 *(0.50 – 1.40)* | 0.75 *(0.44 -1.25)* |
| | Rangi | 1.94 *(0.68 – 5.54)* | 2.54 *(0.94 – 6.89)* | **2.56*** *(1.03 – 6.35)* | **2.02*** *(1.08 – 3.80)* | 1.03 *(0.58 – 1.83)* |
| | Meru | 2.01 *(0.49 – 8.27)* | **4.50*** *(1.07 – 18.81)* | 1.93 *(0.69 – 5.32)* | **2.66*** *(1.22 – 5.83)* | **2.43*** *(1.15 – 5.13)* |
| | Other | 1.25 *(0.66 – 2.37)* | 1.43 *(0.79 – 2.59)* | 1.49 *(0.87 – 2.56)* | **1.58*** *(1.03 – 2.41)* | 1.26 *(0.84 – 1.90)* |
| Livelihood [a] (Maasai only) | Livestock | 1.00 | 1.00 | 1.00 | 1.00 | 1.00 |
| | Farmer | *0.50* *(0.22 – 1.15)* | 0.85 *(0.39 – 1.86)* | 0.76 *(0.36 – 1.62)* | 1.17 *(0.66 – 2.07)* | 1.24 *(0.68 – 2.26)* |
| Age (months) | | **1.24***** *(1.19 – 1.28)* | **1.22*** *(1.18 – 1.27)* | **1.26***** *(1.22 – 1.30)* | **1.38***** *(1.34 – 1.42)* | **1.22***** *(1.20 – 1.25)* |
| Age (months) squared | | **1.00***** *(1.00 –1.00)* | **1.00***** *(1.00 – 1.00)* | **1.00***** *(1.00 –1.00)* | **1.00***** *(1.00 – 1.00)* | **1.00***** *(1.00 –1.00)* |
| Child Sex | Male | 1.00 | 1.00 | 1.00 | 1.00 | 1.00 |
| | Female | 1.10 *(0.79 – 1.54)* | 1.04 ns *(0.75 – 1.46)* | 0.97 *(0.72 – 1.31)* | 1.02 *(0.82 – 1.26)* | 1.13 *(0.94 – 1.36)* |
| Hunger Season | No | 1.00 | 1.00 | 1.00 | 1.00 | 1.00 |
| | Yes | **0.58+** *(0.30 – 1.10)* | 0.80 *(0.46 – 1.40)* | 0.86 *(0.53 – 1.40)* | 0.98 *(0.64 – 1.48)* | 0.91 *(0.58 – 1.44)* |
| Constant | | **3.12**** *(1.46 – 6.64)* | **2.02**** *(1.12 – 4.36)* | 1.32 *(0.71 – 2.43)* | **0.04***** *(0.02 – 0.07)* | **0.17***** *(0.10 – 0.28)* |
| Random Effects *(VPC)* | | | | | | |
| Village Variance Without Ethnicity | | 0.81 *(20%)* | 0.50 *(13%)* | 0.38 *(10%)* | 0.55 *(14%)* | 0.79 *(19%)* |
| Village Variance With Ethnicity | | 0.85 *(20%)* | 0.54 *(14%)* | 0.40 *(10%)* | 0.42 *(11%)* | 0.58 *(15%)* |

+ p<0.1, * p<0.05, ** p<0.01, *** p<0.001
[a] Livelihood parameters are derived from separate models including only Maasai households.

Table S9
Multilevel Logistic Regressions Predicting Subjective Health and Self-Reported Incidence of Specific Illnesses/Symptoms

| | | "Has the Child Ever Had X?" | | | | | | | | | | | |
|---|---|---|---|---|---|---|---|---|---|---|---|---|---|
| Fixed Effects | | Fever (n = 3586) | | Diarrhoea (n = 3586) | | Cough/Flu[b] (n = 2789) | | Pneumonia (n = 3586) | | Malaria[b] (n = 2789) | | Worms[b] (n = 2496) | |
| | | % | Adjusted Odds Ratio (95% CI) | % | Adjusted Odds Ratio (95% CI) | % | Adjusted Odds Ratio (95% CI) | % | Adjusted Odds Ratio (95% CI) | % | Adjusted Odds Ratio (95% CI) | % | Adjusted Odds Ratio (95% CI) |
| Ethnicity | Maasai | 89 | 1.00 | 70 | 1.00 | 89 | 1.00 | 29 | 1.00 | 54 | 1.00 | 15 | 1.00 |
| | Sukuma | 91 | 1.42<br>(0.86 – 2.35) | 76 | 1.56*<br>(1.13 – 2.17) | 93 | 2.14**<br>(1.32 – 3.43) | 17 | 0.85<br>(0.53 – 1.37) | 71 | 1.58+<br>(0.94 – 2.65) | 24 | 1.36<br>(0.78 – 2.39) |
| | Rangi | 91 | 1.17<br>(0.63 – 2.20) | 75 | 1.31<br>(0.87 – 1.97) | 93 | 1.76+<br>(0.95 – 3.26) | 17 | 0.78<br>(0.45 – 1.33) | 77 | 2.08*<br>(1.15 – 3.77) | 25 | 0.97<br>(0.48 – 1.94) |
| | Meru | 87 | 0.64<br>(0.32 – 1.27) | 61 | 0.72<br>(0.46 – 1.14) | 90 | 1.10<br>(0.51 – 2.39) | 29 | 1.18<br>(0.66 – 2.10) | 60 | 1.03<br>(0.53 – 2.05) | 35 | 2.08*<br>(1.01 – 4.30) |
| | Other | 89 | 0.99<br>(0.64 – 1.52) | 75 | 1.39*<br>(1.05 – 1.83) | 91 | 1.48+<br>(0.94 – 2.35) | 18 | 0.86<br>(0.59 – 1.25) | 71 | 1.67*<br>(1.07 – 2.60) | 25 | 1.20<br>(0.69 – 2.08) |
| Livelihood[a] (Maasai only) | Livestock | 89 | 1.00 | 71 | 1.00 | 87 | 1.00 | 28 | 1.00 | 52 | 1.00 | 8 | 1.00 |
| | Farmer | 91 | 1.06<br>(0.54 – 2.11) | 66 | 0.81<br>(0.53 – 1.22) | 92 | 1.53<br>(0.67 – 3.50) | 34 | 1.26<br>(0.83 – 1.90) | 57 | 1.79<br>(0.84 – 3.80) | 22 | 3.55**<br>(1.69 – 7.47) |
| Age (months) | | 1.24***<br>(1.20 – 1.27) | | 1.14***<br>(1.12 – 1.16) | | 1.16***<br>(1.13 – 1.20) | | 1.06***<br>(1.04 – 1.08) | | 1.16***<br>(1.14 – 1.19) | | 1.10***<br>(1.08 – 1.14) | |
| Age (months) squared | | 1.00***<br>(1.00 – 1.00) | | 1.00***<br>(1.00 – 1.00) | | 1.00***<br>(1.00 – 1.00) | | 1.00***<br>(1.00 – 1.00) | | 1.00***<br>(1.00 – 1.00) | | 1.00***<br>(1.00 – 1.00) | |
| Child Sex | Male | 1.00 | | 1.00 | | 1.00 | | 1.00 | | 1.00 | | 1.00 | |
| | Female | 0.73**<br>(0.57 – 0.93) | | 0.90<br>(0.77 – 1.05) | | 0.90<br>(0.68 – 1.19) | | 0.93<br>(0.79 – 1.11) | | 0.85+<br>(0.71 – 1.02) | | 1.22*<br>(1.01 – 1.49) | |
| Hunger Season | No | 1.00 | | 1.00 | | 1.00 | | 1.00 | | 1.00 | | 1.00 | |
| | Yes | 1.15<br>(0.79 – 1.68) | | 1.17<br>(0.91 – 1.51) | | 0.77<br>(0.54 – 1.08) | | 1.00<br>(0.68 – 1.45) | | 0.90<br>(0.56 – 1.44) | | 0.85<br>(0.56 – 1.30) | |
| Constant | | 0.83<br>(0.51 – 1.35) | | 0.47<br>(0.33 – 0.66) | | 1.35<br>(0.82 – 2.21) | | 0.10***<br>(0.06 – 0.17) | | 0.19***<br>(0.12 – 0.32) | | 0.04***<br>(0.02 – 0.08) | |
| Random Effects (VPC) | | | | | | | | | | | | | |
| Village Variance Without Ethnicity | | 0.31 (9%) | | 0.18 (5%) | | 0.14 (4%) | | 0.43 (12%) | | 0.52 (14%) | | 0.25 (8%) | |
| Village Variance With Ethnicity | | 0.25 (7%) | | 0.12 (4%) | | 0.07 (2%) | | 0.37 (10%) | | 0.41 (11%) | | 0.23 (7%) | |

+ p<0.1, * p<0.05, ** p<0.01, *** p<0.001

[a] Livelihood parameters are derived from separate models including only Maasai households.

[b] Survey revisions during the project led to the question "Has the Child Ever Had Cough/Flu?" and "Has the Child Ever Had Malaria?" only being asked in villages 15-56, while the question "Has the Child Ever Had Worms?" was only asked in villages 20-56, leading to lower sample sizes for these comparisons.
